# Hanks-Type Serine/Threonine Protein Kinases and Phosphatases in Bacteria: Roles in Signaling and Adaptation to Various Environments

**DOI:** 10.3390/ijms19102872

**Published:** 2018-09-21

**Authors:** Monika Janczarek, José-María Vinardell, Paulina Lipa, Magdalena Karaś

**Affiliations:** 1 Department of Genetics and Microbiology, Institute of Microbiology and Biotechnology, Faculty of Biology and Biotechnology, Maria Curie-Skłodowska University, Akademicka 19 St., 20-033 Lublin, Poland; paulina.lipa56@gmail.com (P.L.); magdalena.karas@poczta.umcs.lublin.pl (M.K.); 2 Department of Microbiology, Faculty of Biology, University of Sevilla, Avda. Reina Mercedes 6, 41012 Sevilla, Spain; jvinar@us.es

**Keywords:** serine/threonine protein kinase, serine/threonine protein phosphatase, reversible protein phosphorylation, signal transduction, regulatory network, bacterial gene expression

## Abstract

Reversible phosphorylation is a key mechanism that regulates many cellular processes in prokaryotes and eukaryotes. In prokaryotes, signal transduction includes two-component signaling systems, which involve a membrane sensor histidine kinase and a cognate DNA-binding response regulator. Several recent studies indicate that alternative regulatory pathways controlled by Hanks-type serine/threonine kinases (STKs) and serine/threonine phosphatases (STPs) also play an essential role in regulation of many different processes in bacteria, such as growth and cell division, cell wall biosynthesis, sporulation, biofilm formation, stress response, metabolic and developmental processes, as well as interactions (either pathogenic or symbiotic) with higher host organisms. Since these enzymes are not DNA-binding proteins, they exert the regulatory role via post-translational modifications of their protein targets. In this review, we summarize the current knowledge of STKs and STPs, and discuss how these enzymes mediate gene expression in prokaryotes. Many studies indicate that regulatory systems based on Hanks-type STKs and STPs play an essential role in the regulation of various cellular processes, by reversibly phosphorylating many protein targets, among them several regulatory proteins of other signaling cascades. These data show high complexity of bacterial regulatory network, in which the crosstalk between STK/STP signaling enzymes, components of TCSs, and the translational machinery occurs. In this regulation, the STK/STP systems have been proved to play important roles.

## 1. Introduction

How bacteria sense and respond to the environment is a fundamental question of bacterial physiology. The survival of microorganisms in the environment depends on their capacity to quickly respond to and adapt to constantly changing conditions. Bacteria occupy different ecological niches. Many bacteria are able to either exist in a free-living stage or interact with the host organism (e.g., pathogenic and symbiotic bacteria) [1,2]. This adaptive potential is ensured by the ability of bacterial cells to sense and transduce both external and internal signals. Protein kinases and their cognate phosphatases, which participate in signal transduction by catalyzing reversible protein phosphorylation, play essential roles in sensing of the external stimuli [3,4]. Phosphorylation is probably the most prevalent and best characterized post-translational modification, and its biological functions are well documented. It is now clear that this modification is widespread in all three domains of life, Eukarya, Bacteria, and Archaea [5,6]. Similarly to eukaryotes, highly diverse enzymatic families with this type of activity (kinases/phosphatases) have been found in bacteria. These enzymes phosphorylate and dephosphorylate various amino acid residues in proteins, most commonly serine (Ser), threonine (Thr), tyrosine (Tyr), histidine (His), and arginine (Arg) [3,7]. Phosphorylation of these specific amino acids in proteins is an essential component of many signal transduction pathways. In such pathways, in addition to protein kinases and phosphatases, phosphoproteins that “sense” other regulatory proteins play an essential role [8,9]. Thus, phosphorylation can control the activity of target proteins, either directly, by inducing conformational changes in proteins, or indirectly, by regulating protein-protein interactions. 

In bacteria, this large number of protein kinases has been classified into five types. These include: His kinases, Tyr kinases, Arg kinases, Hanks-type Ser/Thr kinases (STKs) (also commonly named eukaryotic-like STKs), and atypical Ser kinases [3]. Recently, Nguyen and others [10] have proposed a new family of protein kinases with a Ser/Thr/Tyr kinase activity, that was previously identified as a family of ATPases. A prototypic member of this family, YdiB from *Bacillus subtilis*, has a unique ATP-binding fold, not found in the known protein kinases. In general, while all types of kinases are widespread in bacteria, some are restricted to only some species (atypical Ser kinases). His kinases and atypical Ser kinases are involved in the regulation of gene expression and the control of metabolism, respectively, whereas Tyr kinases and Hanks-type STKs regulate several aspects of bacterial physiology. Unlike Eukarya, most tyrosine phosphorylation in bacteria is conducted not by Hanks-type kinases, but by non-Hanks-type kinases, which are responsible for most of the tyrosine kinase activity [3,5,7]. Bacterial two-component systems (TCSs), in which a membrane sensor His kinase activates a transcription factor-response regulator in response to a specific signal, play a dominant role in bacterial signaling. However, recent studies have shown that signaling systems composed of STKs and Ser/Thr phosphatases (STPs) also play an important role in bacterial regulatory networks. Even though these systems do not have dedicated transcription factors, they are capable of affecting gene expression [11,12,13]. Recent phosphoproteomic analyses identified numerous (ca. 100) proteins phosphorylated on Ser or Thr residues in both Gram-positive and Gram-negative bacteria, as well as in Archaea, indicating that regulation based on STK/STP enzymes is common in these microorganisms [14,15,16,17]. 

In this review, we focused on the recent findings about STKs, which share structural and functional homology with eukaryotic STKs, and their partner STPs, which play an important role in balancing protein kinase functions. We here discuss their roles in bacterial signaling and physiology (protein phosphorylation and its role in signal transduction in Archaea have been recently reviewed in References [16,17]). To date, essentially more data are available on STKs than on STPs, indicating that partners of these kinases have not yet been analyzed in detail and additional studies must be performed for a comprehensive overview of the role of these proteins in bacterial regulatory networks. 

## 2. Structure and Mechanism of Action of Bacterial STKs and STPs

### 2.1. Structure and Mechanism of Action of Bacterial STKs

In 1988, Hanks et al. [18] defined and described the main family of Ser/Thr/Tyr protein kinases present in eukaryotes. It was initially believed that kinases of this type do not exist in bacteria. However, in 1991, Munoz-Dorato and others characterized the first bacterial STK, Pkn1 from *Myxococcus xanthus* [19]. This enzyme shares a structural similarity with eukaryotic STKs and is required for normal development of *M. xanthus*. Since then, numerous studies have indicated that many bacterial species contain protein kinases that share structural similarities with STKs (Table 1). Consequently, these enzymes have long been referred to as “eukaryotic-like” kinases, despite the lack of evidence that they have been acquired by horizontal transfer of eukaryotic genes. Recently, comprehensive phylostratigraphic analyses of Stancik and coworkers [20] suggested that Hanks-type kinases present in Eukarya, Bacteria, and Archaea share a common evolutionary origin in the lineage leading to the last universal common ancestor (LUCA). Moreover, the authors did not find any evidence of horizontal transfer of genes coding for Hanks-type kinases from Eukarya to Bacteria, strongly supporting a monophyletic origin of all Hanks-type kinases. Consequently, these authors proposed to adopt the term “Hanks-type kinases” as a universal name for this enzymatic family [18,20].

**Figure 1:**
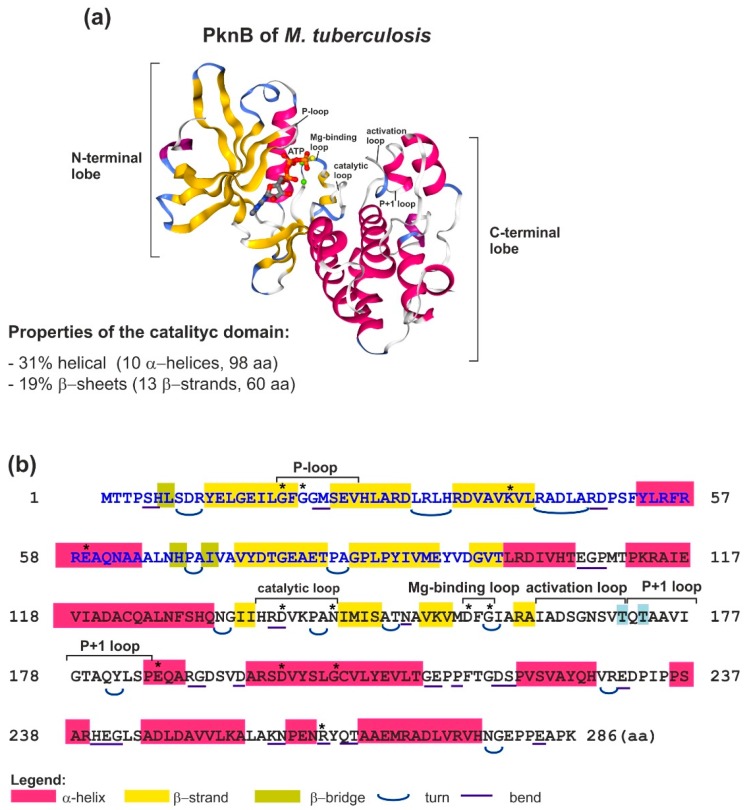
Structure of the catalytic domain of *Mycobacterium tuberculosis* STK PknB. (**a**) Crystal structure of the PknB catalytic domain with an ATP molecule (Protein Data Bank (PDB) accession number 1MRU) [117]. N-terminal and C-terminal lobes as well as individual loops are indicated. α-Helices are shown in pink, β-sheets are in yellow, ATP molecule is shown in red and grey, and Mg^2+^ ions are shown as green spheres. (**b**) Primary amino acid sequence of the 286-residue catalytic domain of PknB. The amino acids (aa) of the N-terminal lobe are blue and aa of the C-terminal lobe are black. Conserved motifs are marked with square brackets, invariant residues are denoted by asterisks, and the phosphorylated Tyr residues in the activation and P+1 loops are shaded in blue.

Hanks-type serine/threonine kinases (STKs) are either membrane or cytoplasmic proteins containing a catalytic domain with 12 specific signatures defined by Hanks. Moreover, STKs possess additional subdomains, which are responsible for the regulation of STK activity or influence their subcellular localization. These kinases can be autophosphorylated. The kinase domains of STKs are typically organized into 12 subdomains that fold into a characteristic two-lobed catalytic core structure with the active site located in a deep cleft formed between the two lobes (Figure 1) [112,113,114]. The N-terminal lobe is involved in the binding and orienting of an ATP molecule (phosphate donor), whereas the C-terminal lobe is responsible for binding to the protein substrate and transfer of the phosphate group. The structural conservation of the catalytic domain in different kinases is remarkable and maintained across eukaryotic and bacterial domains. Despite little sequence homology between members of the superfamily, the kinase catalytic domain can be defined by the presence of specific conserved motifs and 12 nearly invariant residues participating in positioning of the ATP molecule and protein substrate for catalysis [7,112,114]. The activation segment is the most important regulatory element of the kinase. It includes several conserved loop motifs: catalytic, Mg^2+^-binding, activation and P+1 loops (Figure 1). The STK activation occurs by phosphorylation of at least one Ser/Thr residue in the activation loop, and this is achieved by either autophosphorylation or transphosphorylation by another kinase. The activation loop is the most variable region of the activation segment, determines substrate specificity, and is a site of protein-protein interactions that modulate kinase activity. The P+1 loop, which is a critical point of contact between the kinase and its substrate, is a major determinant of the distinct substrate specificity of Ser/Thr and Tyr kinases. In the former, this loop contains a conserved Ser or Thr residue that interacts with the catalytic loop. A glycine-rich P loop plays an important role in the transfer of the phosphoryl group and exchange of ATP/ADP during the catalytic cycle [7]. All these conformational changes allow the transfer of a γ-phosphate group from ATP to the Ser or Thr residue in the protein substrate. The first described structure of bacterial STK was that of *Mycobacterium tuberculosis* PknB (Figure 1) [115,116,117]. The structure of this protein was found to be very similar to that of the mouse cyclic AMP-dependent protein kinase (PKA). The catalytic domain of PknB exhibits the typical two-lobed structure. Structure similarities between these proteins suggest a common activation mechanism shared by eukaryotic and prokaryotic STKs. PknB was crystalized as a dimer, indicating interactions between the opposite sides of the N-terminal lobes of two catalytic domains. The obtained results support a similar model of activation for bacterial STKs. The importance of dimerization in kinase activation was further documented by mutagenesis studies, in which the replacement of conserved amino acid (aa) residues in the N-terminal lobe reduced autophosphorylation and altered substrate specificity [7,118,119]. However, the mechanism by which dimerization results in autophosphorylation remains unknown. One of the proposed hypotheses concerns the formation of asymmetric dimers. As shown for *M. tuberculosis* PknB, dimerization resulting in front-to-front asymmetric dimers enables subunit interactions, in which one monomer functions as an activator of the second monomer (a substrate), thus mimicking a *trans*-autophosphorylation complex [120]. 

Apart from the catalytic domain, many bacterial STKs contain, additional domain(s) that mediate the binding of ligands and/or protein-protein interactions [e.g., penicillin-binding and Ser/Thr kinase-associated repeats (PASTA) and forkhead-associated domains responsible for recognizing phosphothreonine epitopes on proteins (FHA)] [114]. It has been found that the variability in this modular organization of STKs is characteristic of bacteria that have a few different STKs, such as *Myxobacterium*, *Streptomyces coelicolor*, and *Streptococcus pneumoniae*. Among membrane STKs, one class of these enzymes (PASTA-STKs) possesses the extracellular PASTA domain, which is not found in Eukaryotes. Gram-positive bacteria possess at least one STK located in the membrane, which is composed of a cytoplasmic catalytic domain linked by a transmembrane segment to an extracellular domain containing a variable number of PASTA motifs. Importantly, membrane STKs with PASTA motifs play a major role in the regulation of bacterial cell division and morphogenesis. These motifs interact with the peptidoglycan and serve as a regulatory domain of STK kinase activity [7,121,122,123,124]. The PASTA domains were characterized for the first time by crystal structure analyses in the PBP2X protein from *S. pneumoniae*. In this protein, two PASTA repeats with a unique β_3_α topology were found [124]. In the case of *M. tuberculosis*, the extracellular sensor domain of the transmembrane kinase PknD forms a propeller-like circle structure composed of six β-sheet motifs symmetrically arranged around a central pore [125], whereas the kinase PknB contains an extracellular domain that is composed of four linearly organized PASTA repeats [126]. PknB from *Staphylococcus aureus* shows a similar linear organization of its sensor extracellular domain, which contains three PASTA repeats [127]. As has been shown for *B. subtilis* PrkC, the PASTA motifs can interact with the peptidoglycan, a ligand of the STK receptor site [128]. The presence of a ligand has also been found to play a role in the dimerization of these enzymes, as it was reported for PknB from *M. tuberculosis* and PknB from *S. aureus*: in these STKs, binding of peptidoglycan to the PASTA repeats induces dimerization of two kinase molecules, resulting in STK activation [7,126,128]. Thus, PASTA-STKs would behave as membrane receptors able to signal information about the status of the cell wall to their endogenous phosphorylation targets. Several genetic studies indicate that generally genes encoding the PASTA-STKs are located next to and co-transcribed with genes coding for their cognate STPs (e.g., for PknB/PstP of *M. tuberculosis*, PknB/PppL of *S. mutans*, PrkC/PrpC of *B. subtilis*, Stk1/Stp1 of *S. aureus*, Stk1/Stp1 of *B. anthracis*, Stk1/Stp1 of *S. agalactie*, and SP-STK/SP-STP of *S. pyogenes*) (Table 1) [47,52,65,67].

Based on both genomic and proteomic studies, it has been established that various bacterial species harbor more than one STK and, frequently, the number of STKs reflects the complexity of the environments inhabited by these bacterial species [7,20]. Interestingly, mycobacteria have an unusually large repertoire of this type of kinases. For example, 11 STKs have been identified in *M. tuberculosis*; these proteins are involved in various cellular processes, such as growth, development, biofilm formation, antibiotic resistance, primary and secondary metabolism, stress responses, and virulence (Table 1) [129,130,131,132,133]. Further, multiple STKs have been identified and characterized in other microorganisms (e.g., *Anabaena* sp., *Synechocystis* sp., and *M. xanthus*). Using different methods (in vitro kinase assay, mass spectrometry, site-directed mutagenesis, 2D electrophoresis, and phospho-amino acid analysis), target proteins for several individual STKs have been determined (Table 1). Consequently, a large range of substrate-specificity has been described for these kinases, including other STKs. This indicates a high complexity of bacterial regulatory networks, in which these STKs appear to play a major role. 

Interestingly, the great majority of STKs discovered to date are from Gram-positive bacteria, and only a few have been thus far identified in Gram-negative bacteria. In fact, it had been postulated that some bacteria, such as *Escherichia coli*, do not possess STK orthologs. However, recently, Li and others [110] have discovered a putative STK, Stk, that acts as an effector in a strain of enterohemorrhagic *E. coli* (EHEC). This previously unknown effector, which upon translocation to the infected mouse cells efficiently phosphorylates IκBα and activates the NF-κB pathway, induces aggressive host inflammatory response during EHEC infection. In addition, other STK orthologs have also been identified recently in other pathogenic Gram-negative bacteria, such as *Pseudomonas aeruginosa* (PpkA) and *Yersinia pseudotuberculosis* (YpkA) [107,109].

Interestingly, according to genomic analyses, STK orthologs can also be found in non-pathogenic Gram-negative bacteria, e.g., soil bacteria that establish symbiotic interactions with legumes (rhizobia). For instance, three genes encoding putative STKs have been identified in the genome of *Rhizobium leguminosarum* Rt24.2, although protein substrates for these putative STKs are still unknown [111]. 

To summarize, the available data indicate that Gram-positive bacteria possess a higher variety of STKs than Gram-negative bacteria. Among them, *M. tuberculosis* has the highest number of enzymes of this type determined thus far (Table 1).

### 2.2. Structure and Function of Bacterial STPs

The bacterial ability to sense and respond to the changing environmental conditions requires continuous and reversible phosphorylation. Apart from STKs, cognate STPs are engaged in this process [7,13]. However, up to now, less bacterial STPs have been discovered and biochemically characterized than STKs (Table 2). This may be because (i) enzymes of this type have not been of great interest to researchers to date and (ii) the number of STPs present in bacterial cells is considerably smaller than that of STKs. Even in Gram-positive pathogenic bacteria, which have been intensely studied for many years, only a few STPs have been identified so far, independently of the type of ecological niche that they inhabit [134]. Further, in the case of Gram-negative bacteria, only a few examples of these enzymes have been described, and data are especially scarce for the soil bacteria (Table 2). As commented above, this may be partially explained by the fact that, for a long time, proteins with these activities were not of scientific interest since, similarly to TCSs and phosphorelay signal transduction (where both phosphohistidine (His-P) and aspartyl-phosphate groups undergo relatively rapid hydrolysis), they were considered to not be necessary for dephosphorylation of Ser-P- and Thr-P-phosphorylated regulatory proteins. However, phosphorylated Thr and Ser residues are not as labile as His-P, and, therefore, cognate phosphatases are needed to quench the signaling cascades involving cognate STKs [7,13,135]. 

**Figure 2:**
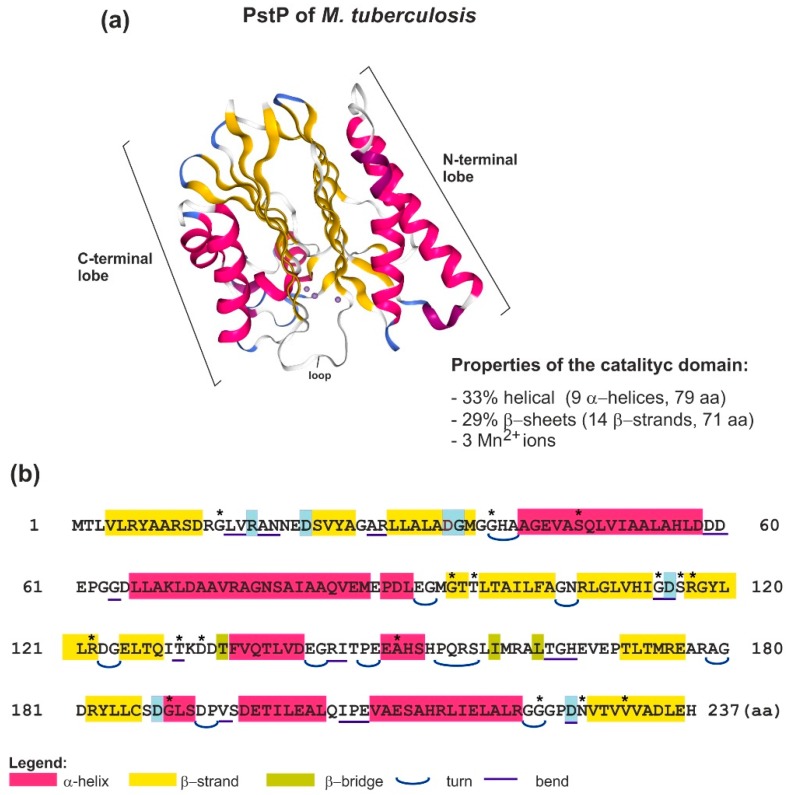
Structure of the catalytic domain of *M. tuberculosis* STP PstP. (**a**) Crystal structure of the PstP catalytic domain with Mn^2+^ ions (PDB accession number 1TXO) [178]. The β-sandwich is represented in yellow, α-helices are represented in pink, and Mn^2+^ ions are shown as purple spheres. N-terminal and C-terminal lobes and the large irregular loop are indicated. (**b**) Primary amino acid sequence of the 237-residue catalytic domain of PstP. Conserved amino acids are denoted by asterisks, and those forming a part of the metal-binding pocket are shaded in blue.

Up to now, four protein phosphatase superfamilies have been identified in Bacteria and Archaea, including phosphoprotein phosphatases (PPPs), metal-dependent phosphatases (PPMs), and conventional and low-molecular-weight protein Tyr phosphatases [12,16,17,35,166]. Enzymes with the STP activity are members of the two structurally different families, PPPs and PPMs. However, a great majority of identified and biochemically characterized STPs belong to the PPM family. Generally, enzymes of the PPP superfamily dephosphorylate Ser-P and Thr-P of the protein substrates. An example of an enzyme with this activity is PrpA from *E. coli* [160]. However, some members of this family, as was confirmed by in vitro studies, show dual-specificity, and can remove phosphate groups not only from Ser-P and Thr-P, but also from Tyr-P (e.g., TpbA from *P. aeruginosa* and PP1-cyano2 from *Microcystis aeruginosa*) [7,35,159,162,167,168]. Serine/threonine protein phosphatases (STPs) belonging to the PPM family can be either Mg^2+^- or Mn^2+^-dependent phosphatases that, although differing in size, share a common conserved catalytic domain consisting of 9–11 signature sequence motifs, in which there are eight conserved aa residues [5,13,16,169,170]. Based on the biochemical properties of bacterial PPM PP2C-type STPs characterized to date, these enzymes preferentially use Mn^2+^ as the metal ion [171]. The N-terminal catalytic domains of all known PP2C STPs share a common core that spans ca. 300 aa residues [172,173,174]. Comparative sequence analysis of the core region of various PP2C STPs revealed the presence of 11 conserved motifs and eight invariant residues (one aspartate (Asp) in motifs 1 and 2, Thr in motif 4, glycine (Gly) in motifs 5 and 6, Asp and Gly in motif 8, and Asp in motif 11) [175,176]. Some PP2C STPs contain additional motifs, motifs 5a and 5b, located between motifs 5 and 6. Depending on the presence of motifs 5a and 5b, the PP2C STPs are subdivided into two subfamilies [171]. Some examples of enzymes from the first subfamily, which lack these motifs, are IcfG from *Synechocystis* sp. PCC 6803, and sporulation-specific phosphatase SpolIE and stress response phosphatases RsbP, RsbU, and RsbX from *B. subtilis* (Table 2). Enzymes belonging to the second subfamily of PPM PP2C contain all signature motifs and are cognate phosphatases of STKs described above. Interestingly, no inhibitors of these phosphatases have been identified thus far. Usually, a specific STP from the PPM family is dedicated to a particular STK, and these proteins are frequently encoded by genes that belong to the same operon. Interestingly, some discrepancies between the numbers of STKs and STPs in individual bacterial species have been observed; the former are usually more numerous than the latter. The most spectacular example is *M. tuberculosis*, which contains 11 STKs and only one STP identified to date [12,35,169]. Bacterial STPs from the PPM family share an essential structural similarity with the human PP2C phosphatase, which is involved in cell differentiation, growth, metabolism, and stress response (Figure 2) [13,177]. The catalytic core domain of human PP2C comprises a central β-sandwich formed by the association of two five-stranded anti-parallel β-sheets surrounded by a pair of anti-parallel α-helices on either side. This spatial arrangement generates a cleft, which acts as a metal center for two metal ions (each metal ion is hexacoordinated by conserved aa and water molecules). This constitutes the active site of the enzyme. The phosphatase activity of PP2C enzymes (dephosphorylation) most probably involves a nucleophilic attack of the phosphorous atom by a metal-activated water nucleophile. This mechanism, which is similar in eukaryotes and bacteria, is ensured by the presence of conserved aa in the active site of these enzymes. The main difference between human PP2C and members of the bacterial PP2C family is the lack of 3 α-helices in the latter (helices 7–9), which are most probably involved in substrate specificity and/or regulation of the human PP2C. Up to date, only a few proteins from the bacterial PPM family have been crystallized (e.g., PstP from *M. tuberculosis*, SaSTP from *Streptococcus agalactiae*, and PphA from *Thermosynechococcus elongatus*) [178,179,180]. The obtained data indicate that the catalytic domains of these bacterial enzymes are structurally nearly identical to the catalytic domain of the human PP2C, with the presence of highly conserved aa residues in the active site (Figure 2). However, a few structural differences were found. These include the absence of histidine (His62) in the active site of bacterial enzymes, and the presence of an additional (third) metal ion in the active site and a loop above the active site, which is most probably involved in the regulation of substrate binding and catalysis [7,13,155]. 

## 3. The Role of STKs and STPs in Bacterial Signaling and Physiology

### 3.1. Interactions of STKs and STPs with Transcriptional Regulators

**Figure 3:**
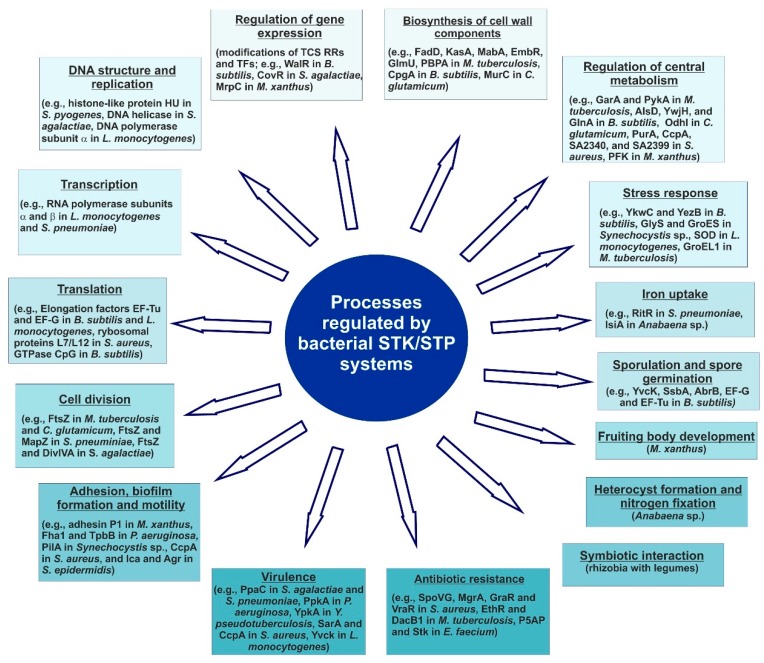
A scheme showing physiological processes regulated by bacterial Hanks-type STKs and STPs in different bacteria.

Analyses of various mutant strains of bacteria lacking Hanks-type serine/threonine kinase (STK) and/or serine/threonine phosphatase (STP) have facilitated the understanding of how these signaling enzymes contribute to the regulation of gene expression in prokaryotes. STK- and STP-mediated gene expression has been proven to be essential for various cellular processes, such as bacterial growth, cell division and morphology, iron transport, secondary metabolite production, antibiotic resistance, virulence, and interactions with plants (Table 1 and Table 2) (Figure 3) [12,15,16,165,166]. 

Although STKs and STPs are not DNA-binding proteins, they mediate gene expression via post-translational modifications of a wide range of protein targets, including TCS response regulators, and key components of the transcriptional and translational machineries. This mechanism ensures an additional level of control of TCS-mediated gene expression, which increases the versatility of bacterial adaptation to changing environmental conditions. As shown for *B. subtilis*, a Gram-positive model bacterium widely used in both basic research and industrial applications, its PrkC kinase and PrpC phosphatase are involved in spore development and biofilm formation [51,181]. The YkwC oxidoreductase is a target of both these enzymes. Phosphorylation of this protein at Ser281 abolishes its activity. Similarly, in the important human pathogen *M. tuberculosis*, STP PstP has been found to be required for accurate cell division and survival [136]. Enzymes with STK and STP activities also affect cell growth, segregation, and virulence in *Streptococcus pyogenes* (SP-STK and SP-STP) and *S. agalactiae* (Stk1 and Stp1) [64,66]. Among other substrates, *S. agalactiae* Stk1 phosphorylates CovR (control of virulence), which is a regulatory component of the TCS CovR/CovS that modulates the expression of over 100 genes associated with virulence, including a gene encoding β-hemolysin [182,183]. Similarly, in *S. pyogenes* (also known as Group A Streptococcus), the CovR/CovS system regulates the expression of a large number of virulence genes, and several products of these genes are SP-STK targets (including CovR) [184,185]. Agarwal and others [67] described that *S. pyogenes* non-polar SP-STP mutants displayed several morphological changes, such as increased bacterial chain length, thickened cell wall, and reduced capsule and hemolysin production. Moreover, SP-STK is involved in the regulation of *S. pyogenes* cell division [67,148]. Similar regulatory relationships between STK/STP and TCS signaling pathways have been found in another human pathogen, *S. pneumoniae*. Transcriptional regulator RitR (Rit stands for “repressor of iron transport”), an *S. agalactiae* CovR homolog, is important for the virulence of this bacterium. The RitR negatively regulates the expression of a gene coding for the iron uptake transporter Piu, and its activity is regulated by reversible phosphorylation by both StkP kinase and PhpP phosphatase [70,186]. 

A high complexity of regulation of gene expression via STK/STP signaling has been observed in *M. tuberculosis*. In this bacterium, 11 STKs (PknA–L) identified thus far recognize and phosphorylate a large range of protein substrates related to various cellular processes, such as cell wall biosynthesis (involved in mycolic acid synthesis (FadD, FabH, KasA, KasB, MabA, and GlmU) and arabinan synthesis (EmbR)), glycogen recycling (GarA), cell division (FtsZ, MurD, and Wag31), heat shock response (GroEL1), and transcription regulation (RshA and SigH) (Table 1) (Figure 3). Moreover, individual Pkn kinases can be substrates of other Pkn enzymes, as has been reported for PknA and PknB, which are each other’s targets. EmbR is a very important protein among the different STK *M. tuberculosis* substrates. This transcriptional regulator from the OmpR-like family plays an important role in cell wall biosynthesis through the regulation of *embC*, *embA*, and *embB* genes encoding three arabinosyltransferases [28,41]. EmbR is a target of several mycobacterial STKs (PknA, PknB, PknH, and PknJ). It has been reported that phosphorylation of this regulatory protein by PknH enhances its ability to bind to the promoter sequences of *embC*, *embA*, and *embB* genes. Interestingly, considering that the only *M. tuberculosis* STP identified thus far is PstP, this protein is thought to dephosphorylate all STKs and their protein substrates. In fact, STKs: PknA, PknB, PknH, and PknJ are among the biochemically confirmed substrates of PstP (Table 2). Thus, PstP, as the sole STP, is extremely important for mycobacterial pathogenesis. These findings illustrate the high complexity of gene regulatory network in this bacterium, in which STKs play a major role. 

Similarly, Stk1 (also called PknB), the only STK identified in *S. aureus* to date, recognizes and phosphorylates a large set of proteins, including two global regulatory proteins, SarA and MgrA (Table 1). The former is involved in the regulation of ca. 100 genes, including the positive regulation of *agrBDCA* expression and negative regulation of the expression of its own gene [187,188]. AgrC and AgrA are the sensor and response proteins, respectively, of a TCS system that senses critical extracellular concentrations of an octapeptide that acts as a quorum sensing signaling molecule in this bacterium. Another global transcriptional regulator, MgrA, is involved in the regulation of the expression of many genes, including a gene encoding a multi-drug efflux pump component, NorA [151,189,190,191,192]. Phosphorylation of MgrA by Stk1 prevents its binding to the *norA* promoter, resulting in increased *norA* transcription [77,190,191].

In contrast with Gram-positive bacteria, considerably less data are available for STKs and STPs in Gram-negative bacteria (Table 1 and Table 2). One of the first identified STKs was that from *M. xanthus*, a Gram-negative soil bacterium that, in response to environmental stress factors, can shift from vegetative growth to the formation of fruiting bodies [193]. Six STKs have been identified in this bacterium (Pkn2, Pkn4, Pkn5, Pkn6, Pkn8, and Pkn14). Among them, Pkn8 and Pkn14 are involved in the regulation of the activity of MrpC, a transcriptional factor essential for the activation of gene expression during fruiting body development. In this regulatory cascade, the membrane kinase Pkn8 phosphorylates the cytoplasmic kinase Pkn14, which subsequently phosphorylates MrpC [92,194]. Phosphorylation of MrpC by Pkn14 prevents its binding to the *mrpC* promoter sequence. Thus, Pkn8/Pkn14-mediated phosphorylation of MrpC represses *mrpC* gene expression during vegetative growth and allows for timely expression of *fruA*, and, in a consequence, fruiting body development in response to environmental stressors. 

Hanks-type serine/threonine kinase and phosphatase (STK and STP enzymes) have also been identified and biochemically characterized in *P. aeruginosa* [107,108,159]. It has been reported that the STK PpkA is a FHA domain-containing protein, whereas the STP PppA is an Mn^2+^-dependent phosphatase belonging to the PP2C family. Both these *P. aeruginosa* enzymes are involved in biofilm formation, tolerance to stress, and virulence. Further, the presence of numerous STKs has been confirmed in *Synechocystis* sp. and *Anabaena* sp. These proteins are engaged in the regulation of cell growth and adaptation to stress conditions (Table 1) [93,94,95,96,99,100,101,102,103,195].

Surprisingly, very little is known about STKs and STPs of the soil and symbiotic nitrogen-fixing bacteria. Recently, several genes encoding putative STKs and STPs have been identified in the genome of *R. leguminosarum* bv. *trifolii* Rt24.2, a nitrogen-fixing symbiont of clover plants (*Trifolium pratense*) [111,165]. Among these genes, *pssZ*, encoding a protein that shares a high similarity with STPs, was identified in the genomic Pss-I region. This region is involved in the synthesis of acidic exopolysaccharide (EPS), which plays an essential role in the symbiotic interactions of many rhizobial strains with their host legumes [196]. The pleiotropic effects of a *pssZ* mutation, including the lack of EPS production, decreased growth and motility, altered cell-surface properties, and failure to infect the host plant, indicate that the STP PssZ is required for several cellular processes, both in the free-living state and during symbiosis.

It is also important to note than many recent studies in bacteria indicate the existence of additional levels of regulation between the different phosphorylation systems, such as cross-phosphorylation of protein kinases. A good example is the bacterium *M. tuberculosis*, in which cross-phosphorylation between various Hanks-type STKs, as well as their phosphorylation by protein Tyr-kinases have been confirmed [3,197]. 

### 3.2. The Role of STKs and STPs in Regulation of Transcription and Protein Biosynthesis 

Apart from many response regulators of various TCSs, different protein components of the transcriptional and translational machineries have been found to be STK and STP substrates (Figure 3). These include: the histone-like protein HU, and the elongation factors EF-G and EF-Tu (Table 1 and Table 2). Similarly to histones in eukaryotes, bacterial histone-like proteins play an essential role in the regulation of DNA replication and transcription, most probably by introducing structural changes in the DNA that facilitate or prevent binding of other regulatory proteins to DNA [198,199,200]. As shown for *E. coli*, the histone-like protein HU regulates the transcription of ca. 8% genes [200]. Recent studies have shown that histone-like proteins are substrates of several STKs and STPs in other bacteria, such as Stk1 from *S. aureus*, Pkn2 from *M. xanthus*, and SP-STK and SP-STP from *S. pyogenes* (Table 1 and Table 2) [89,148,151]. As described for *M. xanthus*, phosphorylation of HUα by Pkn2 prevents its binding to DNA [89]. HUα and HUβ are highly conserved in bacteria, and these proteins function in the regulation of gene expression, acting as heterodimers. Post-translational modifications of histone-like proteins by STKs may affect their binding to DNA or their binding to other transcriptional regulators.

Moreover, STKs and STPs have found to be engaged in the modulation of activity of DNA and RNA polymerases by reversibly phosphorylating these enzymes. For example, RpoA, the α subunit of RNA polymerase, has been identified as a substrate of *S. pneumoniae* StkP (Table 1), suggesting that this kinase may regulate gene expression by controlling the interaction of RpoA with certain transcription factors [74]. In *L. monocytogenes*, PrkA interacts with several proteins that are crucial for replication and transcription, such as the DNA polymerase III subunit α (PolC), the RNA polymerase subunits α and β (RpoA and RpoB), and the recombination protein RecA [105].

Several translation elongation factors, which play an essential role in protein biosynthesis, are substrates of prokaryotic STKs and STPs (Figure 3). Three different factors are engaged in the initiation of the translation elongation step: EF-Tu, EF-Ts, and EF-G. EF-Tu is responsible for delivering aminoacyl-tRNA to the ribosome acceptor site and association with GTP; EF-Ts acts as a guanine nucleotide exchange factor on EF-Tu; and EF-G is an additional GTPase involved in the translocation of mRNA and tRNA [105,157]. In the spore-forming soil bacterium *B. subtilis*, the kinase PrkC and phosphatase PrpC reversibly phosphorylate the elongation factors EF-Tu and EF-G [47,52]. Phosphorylation of EF-Tu prevents its binding to aminoacyl-tRNA and thus inhibits the translation elongation step [157]. EF-Tu is also a substrate of PknB in *M. tuberculosis* [201]. Phosphorylation of this protein reduces its interaction with GTP, increasing resistance to specific antibiotics and decreasing the total level of protein synthesis, which can promote dormancy of *M. tuberculosis* cells. Similarly in *L. monocytogenes*, EF-Tu and EF-G are substrates of the kinase PrkA. Furthermore, Burnside and others [66] showed that in *S. agalactiae*, ATP-dependent DNA and RNA helicases are targets of the kinase Stk1. Collectively, these data suggest that the regulation of the activity of translation elongation factors by phosphorylation in bacteria may serve the purpose of adjusting the level of protein synthesis in response to changing environmental conditions. 

### 3.3. The Role of STKs and STPs in Cell Wall Architecture and Metabolism, Cellular Metabolism, Cell Division, and Adaptation to Stress Conditions 

Several proteins involved in cell envelope and membrane biogenesis have been identified as substrates of prokaryotic STKs and STPs (Table 1 and Table 2) (Figure 3). Among the different bacterial species studied, *M. tuberculosis* stands out because of the large set of STKs involved in this process. Almost all STKs identified in this bacterium (PknA, PknB, PknD, PknE, PknF, PknI, PknK, and PknL) are involved in the biosynthesis of mycolic acids, which are major and specific lipid components of the mycobacterial cell envelope essential for cell survival [202]. These STKs phosphorylate several proteins engaged in the mycolic acid biosynthesis pathway, such as FadD, FabH, KasA, KasB, and MabA. In addition, STKs PknA, PknB, PknH, and PknJ are involved in the regulation of the synthesis of arabinan, an important polysaccharide component of the mycobacterial cell wall. Moreover, PknA and PknB also affect cell division, since they phosphorylate two important proteins involved in this process, FtsZ and FipA (the latter is required for cell division under oxidative stress) [203].

Hanks-type serine/threonine kinases (STKs) with PASTA repeats in the extracellular domain are supposed to sense cell wall-related processes and to be engaged in the regulation of cell wall/envelope biogenesis. This hypothesis has been confirmed for the *B. subtilis* PASTA-containing STK PrkC, which is indispensable for the response of dormant spores and growing cells to peptidoglycan derivatives (muropeptides) [53,128]. A direct and specific interaction between the PASTA domains of this enzyme and muropeptides has indeed been confirmed [204]. In addition, PrkC phosphorylates YvcK, a protein responsible for the adequate localization and function of penicillin-binding protein 1 (PBP1), which is required for cell wall morphogenesis [205]. In this bacterium, all of the Hanks-type STKs characterized to date except for PrkD (PrkA, PrkC, and YabT) are engaged at different stages of the sporulation process (onset, dormancy, germination, and outgrowth) [49,203,206]. While PrkC participates in the initiation of the spore germination process, YabT is involved in inhibition of protein synthesis in the spore during dormancy. Expression of the genes encoding PrkA and YabT strongly increases during sporulation as a result of the action of the spore-specific sigma factors δ^E^ and δ^F^, respectively. PrkA positively affects the expression of a gene encoding another sigma factor important for sporulation, δ^K^ [46,49]. YabT contains DNA-binding domain and is able to phosphorylate the DNA-related RecA protein, RacA (which is involved in the anchoring of DNA to the cell pole), and the global transcriptional regulator AbrB [49,206].

In *S. aureus*, the PASTA-containing Stk1 and its cognate phosphatase Stp1 also play essential roles in the modulation of cell wall structure and susceptibility to cell wall-acting β-lactam antibiotics [150,152]. Moreover, a strain lacking both these proteins exhibited essential defects in cell division, including irregular cell shape and size, and multiple and incomplete septa. This suggests an important role of Stk1 and Stp1 in cell division. Moreover, the Stk/Stp system has been found to participate in numerous metabolic pathways, including glycolysis, the tricarboxylic acid (TCA) cycle, nucleotide metabolism, and synthesis and secretion of some virulence factors (e.g., α-hemolysin) [152]. Similarly, the Stk/Stp system regulates peptidoglycan structure and metabolism in *Enterococcus faecium* (by influencing the crosslinking l,d-transpeptidase pathway) [87].

Interestingly, it has been recently reported that the STP PssZ of the soil bacterium *R. leguminosarum* is involved in the synthesis of EPS, which plays an essential role as a signal molecule in symbiotic interactions with its host plant, clover [165]. A mutant strain lacking a functional PssZ showed, apart from changes in the cell-surface properties, defects in cell division and size. Although protein substrates of this enzyme remain to be identified, this finding suggests that STKs and STPs play important role in the synthesis of envelope components and proper cell division also in Gram-negative bacteria.

In different streptococci (*S. agalactiae*, *S. pyogenes*, *S. pneumoniae*, and *S. mutans*), the regulatory STK/STP system is involved in human pathogenesis and affects cell growth and morphology, the production of hemolysin and fatty acids, and DNA topology (via modulation of the activity of histone-like proteins) [134]. In addition, *S*. *pyogenes* Stp is a secreted phosphatase that, after entering the host cell, mediates its apoptosis [146].

Moreover, bacterial STK/STP systems are involved in the regulation of various metabolic processes (Table 1 and Table 2) (Figure 3). For example, in *M. tuberculosis*, numerous STKs (PknB, PknD, PknE, PknF, PknG, and PknH) regulate glycogen recycling by directly acting on the protein GarA. In *B. subtilis*, PrkC affects central metabolism by phosphorylating AlsD, YwjH, and GlnA, whereas *Corynebacterium glutamicum* kinases PknA, PknB, and PknG are engaged in glutamate metabolism (Table 1). Interestingly, among the various identified and biochemically characterized STKs in the unicellular cyanobacterium *Synechocystis* sp. PCC6803, high specificity of individual proteins to particular cellular processes was found. For example, SpkB participates in motility and the oxidative stress response [95,207,208,209]. A *spkB* mutant exhibits a pronounced hypersensitivity to oxidative stress and severe growth retardation, whereas a *spkD* mutant displays impaired growth at low concentrations of inorganic carbon sources and a *spkG* mutant is sensitive to high salt concentrations [97,99]. Furthermore, among several STKs identified in another cyanobacterium, *Anabaena* sp. PCC7120, which is able to form nitrogen-fixing heterocysts, PknE and PssH are required for heterocyst development and diazotrophic growth, respectively [101,104]. 

## 4. Conclusions

Reversible phosphorylation is a key mechanism that enables bacteria to sense and respond to changing environmental conditions. Signal sensing and transduction in bacteria are conducted by various regulatory systems, including TCSs and STKs/STPs. Many recent studies indicate that pathways controlled by Hanks-type STKs and STPs play an essential role in the regulation of various cellular processes, such as growth and cell division, cell wall biosynthesis, sporulation, biofilm formation, stress response, metabolic and developmental processes, and virulence. STKs and STPs function in the regulation of gene expression by reversibly phosphorylating many protein targets that are involved in bacterial signaling and physiology. However, identification of environmental signals that trigger the signaling cascade and the elucidation of mechanisms that regulate the crosstalk between STK/STP signaling enzymes, elements of TCSs, and the translational machinery require further study. Results of these studies will facilitate understanding of the function of prokaryotic regulatory networks.

## Figures and Tables

**Table 1:** Serine/threonine kinases (STKs) of Gram-positive and Gram-negative bacteria, their substrates, and biological functions.

Species	STK	Substrate	Function	Reference
	PknA	FadD, FabH, KasA, KasB, MabA	Mycolic acid synthesis	[21,22]
*Mycobacterium*		GlmU	Cell wall synthesis	[23]
*tuberculosis*		FtsZ, MurD, Wag31	Cell division	[24,25,26,27]
		PknB	STK, cell signaling	[27]
		EmbR	Arabinan synthesis	[28]
		GroEL1	Heat shock protein	[29]
	PknB	FadD, KasA, KasB, MabA	Mycolic acid synthesis	[21]
		EmbR	Arabinan synthesis	[28]
		GroEL1	Heat shock protein	[29]
		GlmU, PBPA	Cell wall synthesis	[23,30]
		PknA	STK, cell signaling	[27]
		RshA	Anti-sigma factor, oxidative stress response	[31]
		SigH	Alternative sigma factor, oxidative stress response	[31]
		GarA	Glycogen recycling, TCA cycle	[32]
	PknD	FadD, FabH, KasA, KasB, MabA	Mycolic acid synthesis	[21,22,33]
		GarA	Glycogen recycling, TCA cycle	[32]
		GroEL1	Heat shock protein	[29]
		Mmp17	Membrane transporter	[34]
		Rv0516c	Anti-anti-sigma factor	[35]
		Rv1747	ABC transporter	[36]
	PknE	FadD, KasA, KasB, FabH, MabA	Mycolic acid synthesis	[21,22,33]
		GarA	Glycogen recycling, TCA cycle	[32]
		GroEL1	Heat shock protein	[29]
		Rv1747	ABC transporter	[36]
	PknF	FadD, KasA, KasB, FabH	Mycolic acid synthesis	[21,22,37]
		GroEL1	Heat shock protein	[29]
		GarA	Glycogen recycling, TCA cycle	[32]
		EthR	Antibiotic resistance	[38]
		Rv1747	ABC transporter	[37]
	PknG	GarA	Glycogen recycling, TCA cycle, virulence	[39]
	PknH	FadD, FabH, KasA, KasB	Mycolic acid synthesis	[21,22,33]
		GroEL1	Heat shock protein	[29]
		Rv0681	TetR family transcription factor	[40]
		EmbR	Arabinan synthesis, cell wall biosynthesis, virulence	[41]
		DosR	TCS response regulator, oxidative stress tolerance, spore dormancy	[42]
		DacB1	Penicillin-binding protein	[40]
		GarA	Glycogen recycling, TCA cycle	[39]
	PknI	FadD	Mycolic acid synthesis	[21]
	PknJ	EmbR	Arabinan synthesis, cell wall biosynthesis	[41]
		PepE	Peptidase	[43]
		Mma4	Mycolic acid synthesis	[43]
	PknK	FadD	Mycolic acid synthesis	[21]
		VirS	Transcription factor, stress response	[44]
	PknL	FadD, MabA, KasA, KasB	Mycolic acid synthesis	[21,22]
		Rv2175c	DNA-binding protein, cell envelope	[45]
		GroEL1	Heat shock protein	[29]
*Bacillus subtilis*	PrkA	ND	Indirect regulation of transcription factor δ^K^ and regulator ScoC, sporulation	[46]
	PrkC	CpgA	GTPase, peptydoglycan decomposition, late state of ribosome assembly	[47]
		AlsD	Α-acetolactase, central metabolism	[48]
		Icd	Central metabolism	[48]
		YvcK GpsB	Sporulation process, cell growth Cell division protein, sporulation	[49] [49]
		YwjH	Transladolase, central metabolism	[48]
		GlnA	Glutamine synthetase, central metabolism	[48]
		Hpr	Phosphotransferase system kinase	[48]
		WalR	Response regulator of TCS WalRK, cell wall metabolism in stationary phase	[11]
		AbrA	Transcriptional regulator, exoprotease production, competence development and sporulation	[50]
		AbrB	Global transcriptional regulator, transition from exponential to stationary growth phase	[50]
		YkwC	Oxidoreductase	[51]
		EF-G, EF-Tu	Elongation factors, protein translation, spore germination and cell growth	[47,49,52,53]
	PrkD (YbdM)	AbrA	Transcriptional regulator, exoprotease production, competence development	[50]
		AbrB	Global transcriptional regulator, transition from exponential to stationary growth phase	[49]
		DnaC	Helicase, DNA replication, cell growth	[49]
	YabT	SsbA	DNA recombinase, spore development	[54]
		RacA	DNA-related protein, DNA anchoring to the cell pole, sporulation	[49]
		RecA	DNA recombinase, DNA damage repair, sporulation	[49]
		AbrB	Global transcriptional regulator, transition from exponential to stationary growth phase	[49]
		AbrA	Transcriptional regulator, exoprotease production, competence development and sporulation	[49]
		EF-G, EF-Tu	Elongation factors, inhibition of protein translation in spores	[55,56]
	YdiB (Ser/Thr/Tyr)	YdiE	Translation, oxidative stress response	[10]
		MBP *	Human myelin basic protein (artificial substrate)	[10]
*Bacillus anthracis*	PrkC (BA-Stk1)	ND MBP *	Survival within macrophages, virulence Human myelin basic protein (artificial substrate)	[57] [57]
	PrkD	BasPyk	Pyruvate kinase phosphorylation, glycolysis, cell growth and development	[58]
		MBP *	Human myelin basic protein (artificial substrate)	[58]
	PrkG	MBP *	Human myelin basic protein (artificial substrate) STK PrkG involved in cell growth and development	[58]
*Corynebacterium*	PknA	MurC	Cell wall biosynthesis	[59]
*glutamicum*		FtsZ	Cell division	[60]
		OdhI	Glutamate catabolism	[60,61]
		PknG	Soluble STK	[61]
	PknB	FtsZ	Cell division	[60]
		OdhI	Glutamate catabolism	[60,61]
	PknG	OdhI	Glutamate catabolism	[60,61]
	PknL	FtsZ	Cell division	[60]
*Streptococcus*	Stk1	DivIVA	Cell division	[62]
*agalactiae*		CovR	TCS CovRS response regulator, toxin expression, virulence	[63,64]
(Group B		EF-Tu	Elongation factor	[63,64,65]
Streptococcus)		PpaC	Inorganic pyrophosphatase, virulence	[65]
		ND	ATP-dependent DNA i RNA helicases	[66]
*Streptococcus pyogenes*	SP-STK	WalR CovR	TCS WalRK response regulator, cell wall TCS CovRS response regulator, virulence	[67] [67]
(Group A Streptococcus)		SP-HLP, HU	Histone-like protein	[67]
*Streptococcus*	StkP	FtsZ	Cell division, cellular morphogenesis	[68]
*pneumoniae*		DivIVA	Cell division, cellular morphogenesis	[69]
		PpaC	Inorganic pyrophosphatase, virulence	[68]
		RitR	Transcriptional regulator, iron uptake, oxidative stress response	[70]
		MurC	Cell wall biosynthesis	[71]
		ComD	Competence-specific receptor, TCS ComDE	[72]
		RR06	Adhesion, virulence	[73]
		RpoA	RNA polymerase α subunit	[74]
*Streptococcus mutans*	PknB	ND ND	Cell wall metabolism, bacteriocin production, cell wall metabolism, growth, biofilm formation Regulation of Smu2146c, TCSs VicRK and ComDE, oxidative stress tolerance	[75]
*Staphylococcus*	Stk1	SA0498	Ribosomal protein L7/L12	[76]
*aureus*	(PknB)	SA0545	Phosphate acetyltransferase	[76]
(secreted)		SA0731	Enolase	[76]
		SA1359	Elongation factor P	[76]
		SA2340	Glyoxalase	[76]
		SA2399	Fructose biphosphate aldolase	[76]
		MgrA	Global transcriptional regulator, antibiotic resistance	[77]
		SarA	Global transcriptional regulator, virulence	[78]
		SarZ	Oxidative stress response	[79]
		PurA	Purine biosynthesis	[77,78]
		HU	DNA-binding histone-like protein	[77,78]
		CcpA	Catabolite control protein A, carbon metabolism, virulence	[80]
		VraR	Vancomycin-resistance-associated response regulator	[81]
		GraR	TCS GraSR response regulator, antibiotic resistance	[82]
	SpoVG	Transcriptional factor, virulence, antibiotic resistance	[83]
*Staphylococcus epidermidis*	Stk	ND	Polysaccharide intercellular adhesin (PIA) production, biofilm formation, virulence	[84]
*Mycoplasma pneumoniae*	PrkC	ND	Surface protein (adhesin P1), HmW1-3, and MPN474 phosphorylation, adhesion	[85]
*Mycoplasma genitalium*	MG_109	ND	Virulence	[86]
*Enterococcus faecium*	Stk	P_5_AP	Cell signaling, antibiotic resistance, peptydoglycan biosynthesis	[87,88]
*Myxococcus*	Pkn2	HU	Histone-like protein	[89]
*xanthus*	Pkn4	PFK	Glycolysis	[90]
	Pkn5	ND	Soluble STK, cell growth and development, formation of fruiting bodies	[91]
	Pkn6	ND	Transmembrane STK, cell growth and development, formation of fruiting bodies	[91]
	Pkn8	Pkn14	Soluble STK	[92]
		MrpC	Transcription factor, development of fruiting bodies	[92]
	Pkn14	MrpC	Transcription factor, development of fruiting bodies	[92]
*Synechocystis* sp. PCC 6803	SpkA	ND	PilA1, A2, A5, A6, A9, A10 expression, cell motility	[93,94]
		MBP *, casein *, histone *	Artificial substrates	[93,94]
	SpkB	GlyS	Glycyl-tRNA synthetase β-subunit, oxidative stress adaptation	[95]
	SpkC	SpkK	Soluble STK, stress response	[96]
	SpkD	ND	Carbon metabolism, TCA cycle regulation, bacterial growth	[97]
	SpkE	ND	Cell signaling	[98]
	SpkF	SpkC	Membrane-associated STK, stress response	[96]
	SpkG	ND	High salt resistance, stress-mediated signaling	[99]
	SpkK	GroES	Small co-chaperonin	[96]
*Anabaena* sp.	PknA	ND	Optimal growth	[100]
	PknC	ND	Optimal growth	[100]
	PknD	ND	Optimal growth, heterocyst functioning, nitrogen fixation	[101,102,103]
PCC 7120	PknE	ND	Optimal diazotrophic growth, heterocyst differentiation, nitrogen fixation	[101,102,103]
	PknH	ND	Diazotrophic growth, maintaining connections between heterocysts and vegetative cells	[104]
*L.*	PrkA	PolC	DNA Polymerase III α subunit	[105]
*monocytogenes*		RpoA	RNA polymerase α subunit	[105]
		RpoB	RNA polymerase β subunit	[105]
		RecA	Recombinant protein	[105]
		EF-Tu, EF-G	Translation elongation factors	[105]
		Yvck	Cell wall homeostasis, glycerol metabolism, cytosolic survival, virulence	[106]
*Pseudomonas aeruginosa*	PpkA	Fha1	FHA domain-containing protein, hemolysin-coregulated protein 1 (Hcp1) secretion, biofilm formation, virulence, stress tolerance	[107]
		H1 *	Eukaryotic histone H1	[108]
*Yersinia pseudotuberculosis*	YpkA (secreted)	ND	Virulence factor activated by host cell actin, cytoskeleton disruption, inhibition of macrophage function	[109]
*E. coli* (EHEC)	Stk	ND	Virulence	[110]
*Rhizobium leguminosarum*	BAE36_06965 BAE36_16215 BAE36_31125	ND	Optimal cell growth, oxidative stress adaptation	[111]

ND, not determined; * artificial (in vitro model) kinase substrates; TCA cycle—tricarboxylic acid cycle; ABC transporter—ATP-binding cassette transporter; TCS—two-component system.

**Table 2:** Bacterial serine/threonine phosphatases (STPs), their substrates, and biological functions.

Species	STP	Type	Partner kinase	Substrate	Function	References
*Mycobacterium tuberculosis*	PstP	PPM	PknB	PknA	STK, cell signaling, cell growth and division, cell survival	[136,137]
				PknB	STK, cell signaling, cell growth	[137]
				PknH	STK, cell signaling, cell growth	[136]
				PknJ	STK, cell signaling, cell growth	[138]
				PykA	Pyruvate kinase, glycolysis	[138]
				EmbR	Transcriptional regulator of *embCAB* operon	[136]
				ND	S-adenosylhomocysteine hydrolase, homocysteine metabolism	[139]
				PapA5	Cell wall metabolism	[140]
				Rv0019c	FHA-domain-containing protein interacting with FtsZ, GtsQ, and PapA5, cell division	[140]
				PBPA	PBP, cell wall biosynthesis	[30]
*Bacillus*	PrpC	PP2C	PrkC	EF-G, EF-Tu	Translation factor	[47,52]
*subtilis*				PrkC	STK, cell signaling, biofilm formation, sporulation	[47,52]
				CpG	Ribosome-associated GTPase	[47]
				YezB	Stress response	[47]
				HPr kinase	Phosphotransferase system	[141]
	RsbX	PPM	RsbB	RsbS, TsbR	Sigma B regulation, stress response	[142]
	RsbU	PPM	RsbV	RsbV	Sigma B regulation, stress response	[142]
	RsbP	PPM		RsbV	Energy stress response	[142]
	SpoIIE	PPM		SpoIIAA	Anti-anti-sigma factor, sporulation	[143]
*Bacillus anthracis*	PrpC (BA-Stp1)	PP2C	BA-Stk1	PrkC (BA-Stk1)	STK, cell signaling, survival within macrophages, virulence	[144]
				PrkD PrkG	Dual-specificity tyrosine phosphorylation-regulated kinases	[58]
*Streptococcus*	Stp1	PP2C	Stk1	Stk1	STK, cell signaling, cell aggregation	[65]
*agalactiae*				P35	Mn^2+^-dependent inorganic pyrophosphatase	[65]
(group B				PpaC	Inorganic pyrophosphatase	[65]
Streptococcus)				PurA ND	Purine synthesis Hemolysin activity, autolysis, virulence	[65] [65]
*Streptococcus*	PhpP	PP2C	StkP	StkP	STK, cell signaling	[74]
*pneumoniae*				RitR	Transcriptional regulator	[70]
				ComD	Competence-specific receptor, TCS ComDE system	[72]
				MurC	Peptydoglycan synthesis	[71]
				MapZ	Cell division	[145]
				RR06	TCS response regulator	[67,146]
				DivIVA	Cell division	[147]
*Streptococcus*	SP-STP	PP2C	SP-STK	SP-STK	STK, cell signaling	[67,148]
*pyogenes*	(secreted)			SP-HLP ND	Histone-like protein Induction of host cell apoptosis	[148]
*Streptococcus mutans*	PppL	PP2C		PknB	STK, cell signaling	[149]
*Staphylococcus aureus*	Stp1	PP2C	Stk1	Stk1	STK, cell signaling, membrane integrity, cell division, cell wall biosynthesis	[150,151,152]
				GraR	TCS response regulator	[150,151,152]
				MgrA	Global transcriptional regulator	[79]
*Myxococcus xanthus*	Pph1	PP2C	Pkn5	Pkn5	Negative effector of development, vegetative growth and formation of fruiting bodies	[153]
*Enterococcus faecium*	StpA	PP2C	Stk	Stk P_5_AP	STK, cell signaling, antibiotic resistance, peptydoglycan biosynthesis	[85]
				MBP *	Myelin basic protein (artificial substrate)	[85]
*Mycoplasma pneumoniae*	PrpC	PP2C		HPr	Phosphocarrier protein, phosphotransferase system	[154]
*Mycoplasma synoviae*	PrpC	PP2C		ND Phosphopeptides *	Cell signaling Artificial substrates	[155]
*Mycoplasma genitalium*	MG_207	PPM	MG_109	ND	Cell signaling, virulence	[156]
*Listeria*	Stp	PP2C		EF-Tu	Translation factor, protein synthesis regulation	[157]
*monocytogenes*				SOD	Superoxide dismutase, respiratory metabolism	[158]
*Pseudomonas aeruginosa*	PppA	PP2C	PpkA	FHA-1	FHA-domain-containing protein, hemolysin-coregulated protein 1 (Hcp1) secretion	[105]
	TpbA	PPP		TpbB	Dual-specificity Ser/Thr/Tyr kinase, cell motility, biofilm formation	[159]
	Stp1	PP2C		ND	Protein synthesis	[105,159]
*Escherichia coli*	PrpA	PPP		ND Casein, MBP *	Signaling protein misfolding via TCS CpxRA, heat shock response Dual-specificity Ser/Thr/Tyr phosphatase	[160]
*Salmonella enterica* ser. Typhi	PrpZ (Ser/Thr/Tyr)	PP2C		MBP *	Myelin basic protein	[161]
*Synechocystis* sp. PCC 6803	IcfG	PP2C		Slr1856	Carbon metabolism	[162]
	PphA	PPM		PII	Nitrogen assimilation	[163]
*Anabaena* sp. PCC7120	All1758	PP2C		ND	Diazotrophic growth, cell morphology, glycolipid synthesis	[164]
*Rhizobium leguminosarum*	PssZ	PP2C		ND	Cell envelope biogenesis, stress response, motility	[165]

ND, not determined; * artificial (in vitro model) phosphatase substrates; TCS—two-component system; FHA-domain—forkhead-associated domain; PBP—penicillin-binding protein.

## Abbreviations

STK	serine/threonine kinase
STP	serine/threonine phosphatase
PPP	phosphoprotein phosphatase
PPM	metal-dependent phosphatase
TCS	two-component system
LUCA	last universal common ancestral
ATP	adenosine triphosphate
GTP	guanosine triphosphate
Ser	serine
Thr	threonine
Tyr	tyrosine
PASTA	penicillin-binding and Ser/Thr kinase-associated repeat
FHA	forkhead-associated domain
PBP	penicillin-binding protein
EF	elongation factor
MBP	myelin basic protein

## References

[1] DeVinney R., Steele-Mortimer O., Finlay B.B. (2000). Phosphatases and kinases delivered to the host cell by bacterial pathogens. Trends Microbiol..

[2] Rose C.M., Venkateshwaran M., Volkening J.D., Grimsrud P.A., Maeda J., Bailey D.J., Park K., Howes-Podoll M., den Os D., Yeun L.H. (2012). Rapid phosphoproteomic and transcriptomic changes in the rhizobia-legume symbiosis. Mol. Cell Proteomics.

[3] Mijakovic I., Grangeasse C., Turgay K. (2016). Exploring the diversity of protein modifications: Special bacterial phosphorylation systems. FEMS Microbiol. Rev..

[4] Francez-Charlot A., Kaczmarczyk A., Fischer H.M., Vorholt J.A. (2015). The general stress response in *Alphaproteobacteria*. Trends Microbiol..

[5] Kennelly P.J. (2002). Protein kinases and protein phosphatases in prokaryotes: A genomic perspective. FEMS Microbiol. Lett..

[6] Kennelly P.J. (2003). Archaeal protein kinases and protein phosphatases: Insights from genomics and biochemistry. Biochem. J..

[7] Pereira S.F., Goss L., Dworkin J. (2011). Eukaryote-like serine/threonine kinases and phosphatases in bacteria. Microbiol. Mol. Biol. Rev..

[8] Cohen P. (2002). The origins of protein phosphorylation. Nat. Cell Biol..

[9] Kyriakis J.M. (2014). In the beginning, there was protein phosphorylation. J. Biol. Chem..

[10] Nguyen H.A., El Khoury T., Guiral S., Laaberki M.H., Candusso M., Galisson F., Foucher A.E., Kesraoui S., Ballut L., Vallet S. (2017). Expanding the Kinome World: A New Protein Kinase Family Widely Conserved in Bacteria. J. Mol. Biol..

[11] Libby E.A., Goss L.A., Dworkin J. (2015). The Eukaryotic-Like Ser/Thr Kinase PrkC Regulates the Essential WalRK Two-Component System in *Bacillus subtilis*. PLoS Genet..

[12] Brautigan D.L. (2013). Protein Ser/Thr phosphatases—The ugly ducklings of cell signaling. FEBS J..

[13] Shi Y. (2009). Serine/threonine phosphatases: Mechanism through structure. Cell.

[14] Mijakovic I., Macek B. (2012). Impact of phosphoproteomics on studies of bacterial physiology. FEMS Microbiol. Rev..

[15] Dworkin J. (2015). Ser/Thr phosphorylation as a regulatory mechanism in bacteria. Curr. Opin. Microbiol..

[16] Kennelly P.J. (2014). Protein Ser/Thr/Tyr phosphorylation in the Archaea. J. Biol. Chem..

[17] Esser D., Hoffmann L., Pham T.K., Bräsen C., Qiu W., Wright P.C., Albers S.V., Siebers B. (2016). Protein phosphorylation and its role in archaeal signal transduction. FEMS Microbiol. Rev..

[18] Hanks S.K., Quinn A.M., Hunter T. (1988). The protein kinase family: Conserved features and deduced phylogeny of the catalytic domains. Science.

[19] Muñoz-Dorado J., Inouye S., Inouye M. (1991). A gene encoding a protein serine/threonine kinase is required for normal development of *Myxococcus xanthus*, a gram-negative bacterium. Cell.

[20] Stancik I.A., Šestak M.S., Ji B., Axelson-Fisk M., Franjevic D., Jers C., Domazet-Lošo T., Mijakovic I. (2018). Serine/Threonine Protein Kinases from Bacteria, Archaea and Eukarya Share a Common Evolutionary Origin Deeply Rooted in the Tree of Life. J. Mol. Biol..

[21] Molle V., Brown A.K., Besra G.S., Cozzone A.J., Kremer L. (2006). The condensing activities of the *Mycobacterium tuberculosis* type II fatty acid synthase are differentially regulated by phosphorylation. J. Biol. Chem..

[22] Veyron-Churlet R., Zanella-Cléon I., Cohen-Gonsaud M., Molle V., Kremer L. (2010). Phosphorylation of the *Mycobacterium tuberculosis* beta-ketoacyl-acyl carrier protein reductase MabA regulates mycolic acid biosynthesis. J. Biol. Chem..

[23] Parikh A., Verma S.K., Khan S., Prakash B., Nandicoori V.K. (2009). PknB-mediated phosphorylation of a novel substrate, N-acetylglucosamine-1-phosphate uridyltransferase, modulates its acetyltransferase activity. J. Mol. Biol..

[24] Sureka K., Hossain T., Mukherjee P., Chatterjee P., Datta P., Kundu M., Basu J. (2010). Novel role of phosphorylation-dependent interaction between FtsZ and FipA in mycobacterial cell division. PLoS ONE.

[25] Thakur M., Chakraborti P.K. (2006). GTPase activity of mycobacterial FtsZ is impaired due to its transphosphorylation by the eukaryotic-type Ser/Thr kinase, PknA. J. Biol. Chem..

[26] Thakur M., Chakraborti P.K. (2008). Ability of PknA, a mycobacterial eukaryotic-type serine/threonine kinase, to transphosphorylate MurD, a ligase involved in the process of peptidoglycan biosynthesis. Biochem. J..

[27] Kang C.M., Abbott D.W., Park S.T., Dascher C.C., Cantley L.C., Husson R.N. (2005). The *Mycobacterium tuberculosis* serine/threonine kinases PknA and PknB: Substrate identification and regulation of cell shape. Genes Dev..

[28] Sharma K., Gupta M., Krupa A., Srinivasan N., Singh Y. (2006). EmbR, a regulatory protein with ATPase activity, is a substrate of multiple serine/threonine kinases and phosphatase in *Mycobacterium tuberculosis*. FEBS J..

[29] Canova M.J., Kremer L., Molle V. (2009). The *Mycobacterium tuberculosis* GroEL1 chaperone is a substrate of Ser/Thr protein kinases. J. Bacteriol..

[30] Dasgupta A., Datta P., Kundu M., Basu J. (2006). The serine/threonine kinase PknB of *Mycobacterium tuberculosis* phosphorylates PBPA, a penicillin-binding protein required for cell division. Microbiology.

[31] Park S.T., Kang C.M., Husson R.N. (2008). Regulation of the SigH stress response regulon by an essential protein kinase in *Mycobacterium tuberculosis*. Proc. Natl. Acad. Sci. USA.

[32] Villarino A., Duran R., Wehenkel A., Fernandez P., England P., Brodin P., Cole S.T., Zimny-Arndt U., Jungblut P.R., Cerveñansky C. (2005). Proteomic identification of *M. tuberculosis* protein kinase substrates: PknB recruits GarA, a FHA domain-containing protein, through activation loop-mediated interactions. J. Mol. Biol..

[33] Veyron-Churlet R., Molle V., Taylor R.C., Brown A.K., Besra G.S., Zanella-Cléon I., Fütterer K., Kremer L. (2009). The *Mycobacterium tuberculosis* beta-ketoacyl-acyl carrier protein synthase III activity is inhibited by phosphorylation on a single threonine residue. J. Biol. Chem..

[34] Pérez J., Garcia R., Bach H., de Waard J.H., Jacobs W.R., Av-Gay Y., Bubis J., Takiff H.E. (2006). *Mycobacterium tuberculosis* transporter MmpL7 is a potential substrate for kinase PknD. Biochem. Biophys. Res. Commun..

[35] Wright D.P., Ulijasz A.T. (2014). Regulation of transcription by eukaryotic-like serine-threonine kinases and phosphatases in Gram-positive bacterial pathogens. Virulence.

[36] Grundner C., Gay L.M., Alber T. (2005). *Mycobacterium tuberculosis* serine/threonine kinases PknB, PknD, PknE, and PknF phosphorylate multiple FHA domains. Protein Sci..

[37] Molle V., Soulat D., Jault J.M., Grangeasse C., Cozzone A.J., Prost J.F. (2004). Two FHA domains on an ABC transporter, Rv1747, mediate its phosphorylation by PknF, a Ser/Thr protein kinase from *Mycobacterium tuberculosis*. FEMS Microbiol. Lett..

[38] Leiba J., Carrère-Kremer S., Blondiaux N., Dimala M.M., Wohlkönig A., Baulard A., Kremer L., Molle V. (2014). The *Mycobacterium tuberculosis* transcriptional repressor EthR is negatively regulated by Serine/Threonine phosphorylation. Biochem. Biophys. Res. Commun..

[39] O’Hare H.M., Durán R., Cerveñansky C., Bellinzoni M., Wehenkel A.M., Pritsch O., Obal G., Baumgartner J., Vialaret J., Johnsson K. (2008). Regulation of glutamate metabolism by protein kinases in mycobacteria. Mol. Microbiol..

[40] Zheng X., Papavinasasundaram K.G., Av-Gay Y. (2007). Novel substrates of *Mycobacterium tuberculosis* PknH Ser/Thr kinase. Biochem. Biophys. Res. Commun..

[41] Molle V., Kremer L., Girard-Blanc C., Besra G.S., Cozzone A.J., Prost J.F. (2003). An FHA phosphoprotein recognition domain mediates protein EmbR phosphorylation by PknH, a Ser/Thr protein kinase from *Mycobacterium tuberculosis*. Biochemistry.

[42] Chao J.D., Papavinasasundaram K.G., Zheng X., Chávez-Steenbock A., Wang X., Lee G.Q., Av-Gay Y. (2010). Convergence of Ser/Thr and two-component signaling to coordinate expression of the dormancy regulon in *Mycobacterium tuberculosis*. J. Biol. Chem..

[43] Jang J., Stella A., Boudou F., Levillain F., Darthuy E., Vaubourgeix J., Wang C., Bardou F., Puzo G., Gilleron M. (2010). Functional characterization of the *Mycobacterium tuberculosis* serine/threonine kinase PknJ. Microbiology.

[44] Kumar P., Kumar D., Parikh A., Rananaware D., Gupta M., Singh Y., Nandicoori V.K. (2009). The *Mycobacterium tuberculosis* protein kinase K modulates activation of transcription from the promoter of mycobacterial monooxygenase operon through phosphorylation of the transcriptional regulator VirS. J. Biol. Chem..

[45] Canova M.J., Veyron-Churlet R., Zanella-Cleon I., Cohen-Gonsaud M., Cozzone A.J., Becchi M., Kremer L., Molle V. (2008). The *Mycobacterium tuberculosis* serine/threonine kinase PknL phosphorylates Rv2175c: Mass spectrometric profiling of the activation loop phosphorylation sites and their role in the recruitment of Rv2175c. Proteomics.

[46] Yan J., Zou W., Fang J., Huang X., Gao F., He Z., Zhang K., Zhao N. (2015). Eukaryote-like Ser/Thr protein kinase PrkA modulates sporulation via regulating the transcriptional factor σ(K) in *Bacillus subtilis*. Front. Microbiol..

[47] Absalon C., Obuchowski M., Madec E., Delattre D., Holland I.B., Séror S.J. (2009). CpgA, EF-Tu and the stressosome protein YezB are substrates of the Ser/Thr kinase/phosphatase couple, PrkC/PrpC, in *Bacillus subtilis*. Microbiology.

[48] Pietack N., Becher D., Schmidl S.R., Saier M.H., Hecker M., Commichau F.M., Stülke J. (2010). In vitro phosphorylation of key metabolic enzymes from *Bacillus subtilis*: PrkC phosphorylates enzymes from different branches of basic metabolism. J. Mol. Microbiol. Biotechnol..

[49] Pompeo F., Foulquier E., Galinier A. (2016). Impact of Serine/Threonine Protein Kinases on the Regulation of Sporulation in *Bacillus subtilis*. Front. Microbiol..

[50] Kobir A., Poncet S., Bidnenko V., Delumeau O., Jers C., Zouhir S., Grenha R., Nessler S., Noirot P., Mijakovic I. (2014). Phosphorylation of *Bacillus subtilis* gene regulator AbrB modulates its DNA-binding properties. Mol. Microbiol..

[51] Ravikumar V., Shi L., Krug K., Derouiche A., Jers C., Cousin C., Kobir A., Mijakovic I., Macek B. (2014). Quantitative phosphoproteome analysis of *Bacillus subtilis* reveals novel substrates of the kinase PrkC and phosphatase PrpC. Mol. Cell Proteomics.

[52] Gaidenko T.A., Kim T.J., Price C.W. (2002). The PrpC serine-threonine phosphatase and PrkC kinase have opposing physiological roles in stationary-phase *Bacillus subtilis* cells. J. Bacteriol..

[53] Shah I.M., Dworkin J. (2010). Induction and regulation of a secreted peptidoglycan hydrolase by a membrane Ser/Thr kinase that detects muropeptides. Mol. Microbiol..

[54] Bidnenko V., Shi L., Kobir A., Ventroux M., Pigeonneau N., Henry C., Trubuil A., Noirot-Gros M.F., Mijakovic I. (2013). *Bacillus subtilis* serine/threonine protein kinase YabT is involved in spore development via phosphorylation of a bacterial recombinase. Mol. Microbiol..

[55] Derouiche A., Petranovic D., Macek B., Mijakovic I. (2016). *Bacillus subtilis* single-stranded DNA-binding protein SsbA is phosphorylated at threonine 38 by the serine/threonine kinase YabT. Periodicum Biologorum..

[56] Pereira S.F., Gonzalez R.L., Dworkin J. (2015). Protein synthesis during cellular quiescence is inhibited by phosphorylation of a translational elongation factor. Proc. Natl. Acad. Sci. USA.

[57] Bryant-Hudson K.M., Shakir S.M., Ballard J.D. (2011). Autoregulatory characteristics of a *Bacillus anthracis* serine/threonine kinase. J. Bacteriol..

[58] Arora G., Sajid A., Arulanandh M.D., Singhal A., Mattoo A.R., Pomerantsev A.P., Leppla S.H., Maiti S., Singh Y. (2012). Unveiling the novel dual specificity protein kinases in *Bacillus anthracis*: Identification of the first prokaryotic dual specificity tyrosine phosphorylation-regulated kinase (DYRK)-like kinase. J. Biol. Chem..

[59] Fiuza M., Canova M.J., Patin D., Letek M., Zanella-Cléon I., Becchi M., Mateos L.M., Mengin-Lecreulx D., Molle V., Gil J.A. (2008). The MurC ligase essential for peptidoglycan biosynthesis is regulated by the serine/threonine protein kinase PknA in *Corynebacterium glutamicum*. J. Biol. Chem..

[60] Schultz C., Niebisch A., Schwaiger A., Viets U., Metzger S., Bramkamp M., Bott M. (2009). Genetic and biochemical analysis of the serine/threonine protein kinases PknA, PknB, PknG and PknL of *Corynebacterium glutamicum*: Evidence for non-essentiality and for phosphorylation of OdhI and FtsZ by multiple kinases. Mol. Microbiol..

[61] Fiuza M., Canova M.J., Zanella-Cléon I., Becchi M., Cozzone A.J., Mateos L.M., Kremer L., Gil J.A., Molle V. (2008). From the characterization of the four serine/threonine protein kinases (PknA/B/G/L) of *Corynebacterium glutamicum* toward the role of PknA and PknB in cell division. J. Biol. Chem..

[62] Silvestroni A., Jewell K.A., Lin W., Connelly J.E., Ivancic M.M., Tao W.A., Rajagopal L. (2009). Identification of serine/threonine kinase substrates in the human pathogen group B. *Streptococcus*. J. Proteome Res..

[63] Lin W.J., Walthers D., Connelly J.E., Burnside K., Jewell K.A., Kenney L.J., Rajagopal L. (2009). Threonine phosphorylation prevents promoter DNA binding of the Group B *Streptococcus* response regulator CovR. Mol. Microbiol..

[64] Rajagopal L., Vo A., Silvestroni A., Rubens C.E. (2006). Regulation of cytotoxin expression by converging eukaryotic-type and two-component signaling mechanisms in *Streptococcus agalactiae*. Mol. Microbiol..

[65] Rajagopal L., Clancy A., Rubens C.E. (2003). A eukaryotic type serine/threonine kinase and phosphatase in *Streptococcus agalactiae* reversibly phosphorylate an inorganic pyrophosphatase and affect growth, cell segregation, and virulence. J. Biol. Chem..

[66] Burnside K., Lembo A., Harrell M.I., Gurney M., Xue L., BinhTran N.T., Connelly J.E., Jewell K.A., Schmidt B.Z., de los Reyes M. (2011). Serine/threonine phosphatase Stp1 mediates post-transcriptional regulation of hemolysin, autolysis, and virulence of group B *Streptococcus*. J. Biol. Chem..

[67] Agarwal S., Agarwal S., Pancholi P., Pancholi V. (2011). Role of serine/threonine phosphatase (SP-STP) in *Streptococcus pyogenes* physiology and virulence. J. Biol. Chem..

[68] Nováková L., Bezousková S., Pompach P., Spidlová P., Sasková L., Weiser J., Branny P. (2010). Identification of multiple substrates of the StkP Ser/Thr protein kinase in *Streptococcus pneumoniae*. J. Bacteriol..

[69] Giefing C., Jelencsics K.E., Gelbmann D., Senn B.M., Nagy E. (2010). The pneumococcal eukaryotic-type serine/threonine protein kinase StkP co-localizes with the cell division apparatus and interacts with FtsZ in vitro. Microbiology.

[70] Ulijasz A.T., Falk S.P., Weisblum B. (2009). Phosphorylation of the RitR DNA-binding domain by a Ser-Thr phosphokinase: Implications for global gene regulation in the streptococci. Mol. Microbiol..

[71] Falk S.P., Weisblum B. (2013). Phosphorylation of the *Streptococcus pneumoniae* cell wall biosynthesis enzyme MurC by a eukaryotic-like Ser/Thr kinase. FEMS Microbiol. Lett..

[72] Osaki M., Arcondéguy T., Bastide A., Touriol C., Prats H., Trombe M.C. (2009). The StkP/PhpP signaling couple in *Streptococcus pneumoniae*: Cellular organization and physiological characterization. J. Bacteriol..

[73] Agarwal S., Vasudhev S., DeOliveira R.B., Ram S. (2014). Inhibition of the classical pathway of complement by meningococcal capsular polysaccharides. J. Immunol..

[74] Nováková L., Sasková L., Pallová P., Janecek J., Novotná J., Ulrych A., Echenique J., Trombe M.C., Branny P. (2005). Characterization of a eukaryotic type serine/threonine protein kinase and protein phosphatase of *Streptococcus pneumoniae* and identification of kinase substrates. FEBS J..

[75] Banu L.D., Conrads G., Rehrauer H., Hussain H., Allan E., van der Ploeg J.R. (2010). The *Streptococcus mutans* serine/threonine kinase, PknB, regulates competence development, bacteriocin production, and cell wall metabolism. Infect. Immun..

[76] Lomas-Lopez R., Paracuellos P., Riberty M., Cozzone A.J., Duclos B. (2007). Several enzymes of the central metabolism are phosphorylated in *Staphylococcus aureus*. FEMS Microbiol. Lett..

[77] Truong-Bolduc Q.C., Ding Y., Hooper D.C. (2008). Posttranslational modification influences the effects of MgrA on *norA* expression in *Staphylococcus aureus*. J. Bacteriol..

[78] Didier J.P., Cozzone A.J., Duclos B. (2010). Phosphorylation of the virulence regulator SarA modulates its ability to bind DNA in *Staphylococcus aureus*. FEMS Microbiol. Lett..

[79] Sun F., Ding Y., Ji Q., Liang Z., Deng X., Wong C.C., Yi C., Zhang L., Xie S., Alvarez S. (2012). Protein cysteine phosphorylation of SarA/MgrA family transcriptional regulators mediates bacterial virulence and antibiotic resistance. Proc. Natl. Acad. Sci. USA.

[80] Leiba J., Hartmann T., Cluzel M.E., Cohen-Gonsaud M., Delolme F., Bischoff M., Molle V. (2012). A novel mode of regulation of the *Staphylococcus aureus* catabolite control protein A (CcpA) mediated by Stk1 protein phosphorylation. J. Biol. Chem..

[81] Canova M.J., Baronian G., Brelle S., Cohen-Gonsaud M., Bischoff M., Molle V. (2014). A novel mode of regulation of the *Staphylococcus aureus* Vancomycin-resistance-associated response regulator VraR mediated by Stk1 protein phosphorylation. Biochem. Biophys. Res. Commun..

[82] Fridman M., Williams G.D., Muzamal U., Hunter H., Siu K.W., Golemi-Kotra D. (2013). Two unique phosphorylation-driven signaling pathways crosstalk in *Staphylococcus aureus* to modulate the cell-wall charge: Stk1/Stp1 meets GraSR. Biochemistry.

[83] Bischoff M., Brelle S., Minatelli S., Molle V. (2016). Stk1-mediated phosphorylation stimulates the DNA-binding properties of the *Staphylococcus aureus* SpoVG transcriptional factor. Biochem. Biophys. Res. Commun..

[84] Liu Q., Fan J., Niu C., Wang D., Wang J., Wang X., Villaruz A.E., Li M., Otto M., Gao Q. (2011). The Eukaryotic-Type Serine/Threonine Protein Kinase Stk Is Required for Biofilm Formation and Virulence in *Staphylococcus epidermidis*. PLoS ONE.

[85] Schmidl S.R., Gronau K., Hames C., Busse J., Becher D., Hecker M., Stülke J. (2010). The stability of cytadherence proteins in *Mycoplasma pneumoniae* requires activity of the protein kinase PrkC. Infect. Immun..

[86] Fraser C.M., Gocayne J.D., White O., Adams M.D., Clayton R.A., Fleischmann R.D., Bult C.J., Kerlavage A.R., Sutton G., Kelley J.M. (1995). The minimal gene complement of *Mycoplasma genitalium*. Science.

[87] Sacco E., Cortes M., Josseaume N., Rice L.B., Mainardi J.L., Arthur M. (2014). Serine/threonine protein phosphatase-mediated control of the peptidoglycan cross-linking l,d-transpeptidase pathway in *Enterococcus faecium*. mBio.

[88] Desbonnet C., Tait-Kamradt A., Garcia-Solache M., Dunman P., Coleman J., Arthur M., Rice L.B. (2016). Involvement of the Eukaryote-Like Kinase-Phosphatase System and a Protein That Interacts with Penicillin-Binding Protein 5 in Emergence of Cephalosporin Resistance in Cephalosporin-Sensitive Class A Penicillin-Binding Protein Mutants in *Enterococcus faecium*. mBio.

[89] Udo H., Lam C.K., Mori S., Inouye M., Inouye S. (2000). Identification of a substrate for Pkn2, a protein Ser/Thr kinase from *Myxococcus xanthus* by a novel method for substrate identification. J. Mol. Microbiol. Biotechnol..

[90] Nariya H., Inouye S. (2002). Activation of 6-phosphofructokinase via phosphorylation by Pkn4, a protein Ser/Thr kinase of *Myxococcus xanthus*. Mol. Microbiol..

[91] Zhang W., Inouye M., Inouye S. (1996). Reciprocal regulation of the differentiation of *Myxococcus xanthus* by Pkn5 and Pkn6, eukaryotic-like Ser/Thr protein kinases. Mol. Microbiol..

[92] Nariya H., Inouye S. (2005). Identification of a protein Ser/Thr kinase cascade that regulates essential transcriptional activators in *Myxococcus xanthus* development. Mol. Microbiol..

[93] Kamei A., Yuasa T., Orikawa K., Geng X.X., Ikeuchi M. (2001). A eukaryotic-type protein kinase, SpkA, is required for normal motility of the unicellular *Cyanobacterium synechocystis* sp. strain PCC 6803. J. Bacteriol..

[94] Panichkin V.B., Arakawa-Kobayashi S., Kanaseki T., Suzuki I., Los D.A., Shestakov S.V., Murata N. (2006). Serine/threonine protein kinase SpkA in *Synechocystis* sp. strain PCC 6803 is a regulator of expression of three putative *pilA* operons, formation of thick pili, and cell motility. J. Bacteriol..

[95] Mata-Cabana A., García-Domínguez M., Florencio F.J., Lindahl M. (2012). Thiol-based redox modulation of a cyanobacterial eukaryotic-type serine/threonine kinase required for oxidative stress tolerance. Antioxid. Redox Signal.

[96] Zorina A., Stepanchenko N., Novikova G.V., Sinetova M., Panichkin V.B., Moshkov I.E., Zinchenko V.V., Shestakov S.V., Suzuki I., Murata N. (2011). Eukaryotic-like Ser/Thr protein kinases SpkC/F/K are involved in phosphorylation of GroES in the *Cyanobacterium synechocystis*. DNA Res..

[97] Laurent S., Jang J., Janicki A., Zhang C.C., Bédu S. (2008). Inactivation of *spkD*, encoding a Ser/Thr kinase, affects the pool of the TCA cycle metabolites in *Synechocystis* sp. strain PCC 6803. Microbiology.

[98] Zorina A.A., Bedbenov V.S., Novikova G.V., Panichkin V.B., Los D.A. (2014). Involvement of serine/threonine protein kinases in cold stress response in the cyanobacterium *Synechocystis* sp. PCC 6803: Functional characterization of a protein kinase Spke. Mol. Biol. (Mosk).

[99] Liang C., Zhang X., Chi X., Guan X., Li Y., Qin S., Shao H.B. (2011). Serine/threonine protein kinase SpkG is a candidate for high salt resistance in the unicellular cyanobacterium *Synechocystis* sp. PCC 6803. PLoS ONE.

[100] Gonzalez L., Phalip V., Zhang C.C. (2001). Characterization of PknC, a Ser/Thr kinase with broad substrate specificity from the cyanobacterium *Anabaena* sp. strain PCC 7120. Eur. J. Biochem..

[101] Saha S.K., Golden J.W. (2011). Overexpression of *pknE* blocks heterocyst development in *Anabaena* sp. strain PCC 7120. J. Bacteriol..

[102] Zhang C.C., Libs L. (1998). Cloning and characterization of the *pknD* gene encoding an eukaryotic-type protein kinase in the cyanobacterium *Anabaena* sp. PCC7120. Mol. Gen. Genet..

[103] Zhang C.C., Friry A., Peng L. (1998). Molecular and genetic analysis of two closely linked genes that encode, respectively, a protein phosphatase 1/2A/2B homolog and a protein kinase homolog in the cyanobacterium *Anabaena* sp. strain PCC 7120. J. Bacteriol..

[104] Ehira S., Ohmori M. (2012). The *pknH* gene restrictively expressed in heterocysts is required for diazotrophic growth in the cyanobacterium *Anabaena* sp. strain PCC 7120. Microbiology.

[105] Lima A., Durán R., Schujman G.E., Marchissio M.J., Portela M.M., Obal G., Pritsch O., de Mendoza D., Cerveñansky C. (2011). Serine/threonine protein kinase PrkA of the human pathogen *Listeria monocytogenes*: Biochemical characterization and identification of interacting partners through proteomic approaches. J. Proteomics.

[106] Pensinger D.A., Boldon K.M., Chen G.Y., Vincent W.J., Sherman K., Xiong M., Schaenzer A.J., Forster E.R., Coers J., Striker R. (2016). The *Listeria monocytogenes* PASTA kinase PrkA and its substrate YvcK are required for cell wall homeostasis, metabolism, and virulence. PLoS Pathog..

[107] Mougous J.D., Gifford C.A., Ramsdell T.L., Mekalanos J.J. (2007). Threonine phosphorylation post-translationally regulates protein secretion in *Pseudomonas aeruginosa*. Nat. Cell. Biol..

[108] Mukhopadhyay S., Kapatral V., Xu W., Chakrabarty A.M. (1999). Characterization of a Hank’s type serine/threonine kinase and serine/threonine phosphoprotein phosphatase in *Pseudomonas aeruginosa*. J. Bacteriol..

[109] Juris S.J., Rudolph A.E., Huddler D., Orth K., Dixon J.E. (2000). A distinctive role for the *Yersinia* protein kinase: Actin binding, kinase activation, and cytoskeleton disruption. Proc. Natl. Acad. Sci. USA.

[110] Li T., Li Z., Chen F., Liu X., Ning N., Huang J., Wang H. (2017). Eukaryotic-like Kinase Expression in Enterohemorrhagic *Escherichia coli*: Potential for Enhancing Host Aggressive Inflammatory Response. J. Infect. Dis..

[111] Rachwał K., Matczyńska E., Janczarek M. (2015). Transcriptome profiling of a *Rhizobium leguminosarum* bv. *trifolii rosR* mutant reveals the role of the transcriptional regulator RosR in motility, synthesis of cell-surface components, and other cellular processes. BMC Genomics.

[112] Hanks S.K., Hunter T. (1995). Protein kinases 6. The eukaryotic protein kinase superfamily: Kinase (catalytic) domain structure and classification. FASEB J..

[113] Kornev A.P., Taylor S.S. (2010). Defining the conserved internal architecture of a protein kinase. Biochim. Biophys. Acta.

[114] Krupa A., Srinivasan N. (2005). Diversity in domain architectures of Ser/Thr kinases and their homologues in prokaryotes. BMC Genomics.

[115] Ortiz-Lombardía M., Pompeo F., Boitel B., Alzari P.M. (2003). Crystal structure of the catalytic domain of the PknB serine/threonine kinase from *Mycobacterium tuberculosis*. J. Biol. Chem..

[116] Young K.D. (2003). Bacterial shape. Mol. Microbiol..

[117] Young T.A., Delagoutte B., Endrizzi J.A., Falick A.M., Alber T. (2003). Structure of *Mycobacterium tuberculosis* PknB supports a universal activation mechanism for Ser/Thr protein kinases. Nat. Struct. Biol..

[118] Greenstein A.E., Echols N., Lombana T.N., King D.S., Alber T. (2007). Allosteric activation by dimerization of the PknD receptor Ser/Thr protein kinase from *Mycobacterium tuberculosis*. J. Biol. Chem..

[119] Greenstein A.E., Grundner C., Echols N., Gay L.M., Lombana T.N., Miecskowski C.A., Pullen K.E., Sung P.Y., Alber T. (2005). Structure/function studies of Ser/Thr and Tyr protein phosphorylation in *Mycobacterium tuberculosis*. J. Mol. Microbiol. Biotechnol..

[120] Mieczkowski C., Iavarone A.T., Alber T. (2008). Auto-activation mechanism of the *Mycobacterium tuberculosis* PknB receptor Ser/Thr kinase. EMBO J..

[121] Pérez J., Castañeda-García A., Jenke-Kodama H., Müller R., Muñoz-Dorado J. (2008). Eukaryotic-like protein kinases in the prokaryotes and the myxobacterial kinome. Proc. Natl. Acad. Sci. USA.

[122] Petrícková K., Petrícek M. (2003). Eukaryotic-type protein kinases in *Streptomyces coelicolor*: Variations on a common theme. Microbiology.

[123] Yeats C., Finn R.D., Bateman A. (2002). The PASTA domain: A beta-lactam-binding domain. Trends Biochem. Sci..

[124] Dessen A., Mouz N., Gordon E., Hopkins J., Dideberg O. (2001). Crystal structure of PBP2x from a highly penicillin-resistant *Streptococcus pneumoniae* clinical isolate: A mosaic framework containing 83 mutations. J. Biol. Chem..

[125] Good M.C., Greenstein A.E., Young T.A., Ng H.L., Alber T. (2004). Sensor domain of the *Mycobacterium tuberculosis* receptor Ser/Thr protein kinase, PknD, forms a highly symmetric beta propeller. J. Mol. Biol..

[126] Barthe P., Mukamolova G.V., Roumestand C., Cohen-Gonsaud M. (2010). The structure of PknB extracellular PASTA domain from *Mycobacterium tuberculosis* suggests a ligand-dependent kinase activation. Structure.

[127] Paracuellos P., Ballandras A., Robert X., Kahn R., Hervé M., Mengin-Lecreulx D., Cozzone A.J., Duclos B., Gouet P. (2010). The extended conformation of the 2.9-Å crystal structure of the three-PASTA domain of a Ser/Thr kinase from the human pathogen *Staphylococcus aureus*. J. Mol. Biol..

[128] Shah I.M., Laaberki M.H., Popham D.L., Dworkin J. (2008). A eukaryotic-like Ser/Thr kinase signals bacteria to exit dormancy in response to peptidoglycan fragments. Cell.

[129] Cozzone A.J. (2005). Role of protein phosphorylation on serine/threonine and tyrosine in the virulence of bacterial pathogens. J. Mol. Microbiol. Biotechnol..

[130] Kristich C.J., Wells C.L., Dunny G.M. (2007). A eukaryotic-type Ser/Thr kinase in *Enterococcus faecalis* mediates antimicrobial resistance and intestinal persistence. Proc. Natl. Acad. Sci. USA.

[131] Wehenkel A., Bellinzoni M., Graña M., Duran R., Villarino A., Fernandez P., Andre-Leroux G., England P., Takiff H., Cerveñansky C. (2008). Mycobacterial Ser/Thr protein kinases and phosphatases: Physiological roles and therapeutic potential. Biochim. Biophys. Acta.

[132] Molle V., Kremer L. (2010). Division and cell envelope regulation by Ser/Thr phosphorylation: *Mycobacterium* shows the way. Mol. Microbiol..

[133] Ohlsen K., Donat S. (2010). The impact of serine/threonine phosphorylation in *Staphylococcus aureus*. Int. J. Med. Microbiol..

[134] Sajid A., Arora G., Singhal A., Kalia V.C., Singh Y. (2015). Protein Phosphatases of Pathogenic Bacteria: Role in Physiology and Virulence. Annu. Rev. Microbiol..

[135] Stock J.B., Stock A.M., Mottonen J.M. (1990). Signal transduction in bacteria. Nature.

[136] Sharma A.K., Arora D., Singh L.K., Gangwal A., Sajid A., Molle V., Singh Y., Nandicoori V.K. (2016). Serine/Threonine Protein Phosphatase PstP of *Mycobacterium tuberculosis* Is Necessary for Accurate Cell Division and Survival of Pathogen. J. Biol. Chem..

[137] Boitel B., Ortiz-Lombardía M., Durán R., Pompeo F., Cole S.T., Cerveñansky C., Alzari P.M. (2003). PknB kinase activity is regulated by phosphorylation in two Thr residues and dephosphorylation by PstP, the cognate phospho-Ser/Thr phosphatase, in *Mycobacterium tuberculosis*. Mol. Microbiol..

[138] Arora G., Sajid A., Gupta M., Bhaduri A., Kumar P., Basu-Modak S., Singh Y. (2010). Understanding the role of PknJ in *Mycobacterium tuberculosis*: Biochemical characterization and identification of novel substrate pyruvate kinase A. PLoS ONE.

[139] Singhal A., Arora G., Sajid A., Maji A., Bhat A., Virmani R., Upadhyay S., Nandicoori V.K., Sengupta S., Singh Y. (2013). Regulation of homocysteine metabolism by *Mycobacterium tuberculosis* S-adenosylhomocysteine hydrolase. Sci. Rep..

[140] Gupta M., Sajid A., Arora G., Tandon V., Singh Y. (2009). Forkhead-associated domain-containing protein Rv0019c and polyketide-associated protein PapA5, from substrates of serine/threonine protein kinase PknB to interacting proteins of *Mycobacterium tuberculosis*. J. Biol. Chem..

[141] Singh K.D., Halbedel S., Görke B., Stülke J. (2007). Control of the phosphorylation state of the HPr protein of the phosphotransferase system in *Bacillus subtilis*: Implication of the protein phosphatase PrpC. J. Mol. Microbiol. Biotechnol..

[142] Yang X., Kang C.M., Brody M.S., Price C.W. (1996). Opposing pairs of serine protein kinases and phosphatases transmit signals of environmental stress to activate a bacterial transcription factor. Genes Dev..

[143] Obuchowski M., Madec E., Delattre D., Boël G., Iwanicki A., Foulger D., Séror S.J. (2000). Characterization of PrpC from *Bacillus subtilis*, a member of the PPM phosphatase family. J. Bacteriol..

[144] Shakir S.M., Bryant K.M., Larabee J.L., Hamm E.E., Lovchik J., Lyons C.R., Ballard J.D. (2010). Regulatory Interactions of a Virulence-Associated Serine/Threonine Phosphatase-Kinase Pair in *Bacillus anthracis*. J. Bacteriol..

[145] Fleurie A., Lesterlin C., Manuse S., Zhao C., Cluzel C., Lavergne J.P., Franz-Wachtel M., Macek B., Combet C., Kuru E. (2014). MapZ marks the division sites and positions FtsZ rings in *Streptococcus pneumoniae*. Nature.

[146] Agarwal S., Agarwal S., Pancholi P., Pancholi V. (2012). Strain-specific regulatory role of eukaryote-like serine/threonine phosphatase in pneumococcal adherence. Infect. Immun..

[147] Beilharz K., Nováková L., Fadda D., Branny P., Massidda O., Veening J.W. (2012). Control of cell division in *Streptococcus pneumoniae* by the conserved Ser/Thr protein kinase StkP. Proc. Natl. Acad. Sci. USA.

[148] Jin H., Pancholi V. (2006). Identification and biochemical characterization of a eukaryotic-type serine/threonine kinase and its cognate phosphatase in *Streptococcus pyogenes*: Their biological functions and substrate identification. J. Mol. Biol..

[149] Hussain H., Branny P., Allan E. (2006). A eukaryotic-type serine/threonine protein kinase is required for biofilm formation, genetic competence, and acid resistance in *Streptococcus mutans*. J. Bacteriol..

[150] Beltramini A.M., Mukhopadhyay C.D., Pancholi V. (2009). Modulation of cell wall structure and antimicrobial susceptibility by a *Staphylococcus aureus* eukaryote-like serine/threonine kinase and phosphatase. Infect. Immun..

[151] Burnside K., Lembo A., de Los Reyes M., Iliuk A., Binhtran N.T., Connelly J.E., Lin W.J., Schmidt B.Z., Richardson A.R., Fang F.C. (2010). Regulation of hemolysin expression and virulence of *Staphylococcus aureus* by a serine/threonine kinase and phosphatase. PLoS ONE.

[152] Liebeke M., Meyer H., Donat S., Ohlsen K., Lalk M. (2010). A metabolomic view of *Staphylococcus aureus* and its Ser/Thr kinase and phosphatase deletion mutants: Involvement in cell wall biosynthesis. Chem. Biol..

[153] Treuner-Lange A., Ward M.J., Zusman D.R. (2001). Pph1 from *Myxococcus xanthus* is a protein phosphatase involved in vegetative growth and development. Mol. Microbiol..

[154] Halbedel S., Busse J., Schmidl S.R., Stülke J. (2006). Regulatory protein phosphorylation in *Mycoplasma pneumoniae*. A PP2C-type phosphatase serves to dephosphorylate HPr (Ser-P.). J. Biol. Chem..

[155] Menegatti A.C., Vernal J., Terenzi H. (2015). The unique serine/threonine phosphatase from the minimal bacterium *Mycoplasma synoviae*: Biochemical characterization and metal dependence. J. Biol. Inorg. Chem..

[156] Martinez M.A., Das K., Saikolappan S., Materon L.A., Dhandayuthapani S. (2013). A serine/threonine phosphatase encoded by MG_207 of *Mycoplasma genitalium* is critical for its virulence. BMC Microbiol..

[157] Archambaud C., Gouin E., Pizarro-Cerda J., Cossart P., Dussurget O. (2005). Translation elongation factor EF-Tu is a target for Stp, a serine-threonine phosphatase involved in virulence of *Listeria monocytogenes*. Mol. Microbiol..

[158] Archambaud C., Nahori M.A., Pizarro-Cerda J., Cossart P., Dussurget O. (2006). Control of *Listeria* superoxide dismutase by phosphorylation. J. Biol. Chem..

[159] Ueda A., Wood T.K. (2009). Connecting quorum sensing, c-di-GMP, pel polysaccharide, and biofilm formation in *Pseudomonas aeruginosa* through tyrosine phosphatase TpbA (PA3885). PLoS Pathog..

[160] Missiakas D., Raina S. (1997). Signal transduction pathways in response to protein misfolding in the extracytoplasmic compartments of *E. coli*: Role of two new phosphoprotein phosphatases PrpA and PrpB. EMBO J..

[161] Lai S.M., Le Moual H. (2005). PrpZ, a *Salmonella enterica* serovar Typhi serine/threonine protein phosphatase 2C with dual substrate specificity. Microbiology.

[162] Shi L., Bischoff K.M., Kennelly P.J. (1999). The *icfG* gene cluster of *Synechocystis* sp. strain PCC 6803 encodes an Rsb/Spo-like protein kinase, protein phosphatase, and two phosphoproteins. J. Bacteriol..

[163] Kloft N., Rasch G., Forchhammer K. (2005). Protein phosphatase PphA from *Synechocystis* sp. PCC 6803: The physiological framework of PII-P dephosphorylation. Microbiology.

[164] Tom S.K., Callahan S.M. (2012). The putative phosphatase All1758 is necessary for normal growth, cell size and synthesis of the minor heterocyst-specific glycolipid in the cyanobacterium *Anabaena* sp. strain PCC 7120. Microbiology.

[165] Lipa P., Vinardell J.M., Kopcińska J., Zdybicka-Barabas A., Janczarek M. (2018). Mutation in the *pssZ* Gene Negatively Impacts Exopolysaccharide Synthesis, Surface Properties, and Symbiosis of *Rhizobium leguminosarum* bv. *trifolii* with Clover. Genes (Basel).

[166] Burnside K., Rajagopal L. (2012). Regulation of prokaryotic gene expression by eukaryotic-like enzymes. Curr. Opin. Microbiol..

[167] Shi L., Carmichael W.W. (1997). *pp1-cyano2*, a protein serine/threonine phosphatase 1 gene from the cyanobacterium *Microcystis aeruginosa* UTEX 2063. Arch. Microbiol..

[168] Shi L., Carmichael W.W., Kennelly P.J. (1999). Cyanobacterial PPP family protein phosphatases possess multifunctional capabilities and are resistant to microcystin-LR. J. Biol. Chem..

[169] Bork P., Brown N.P., Hegyi H., Schultz J. (1996). The protein phosphatase 2C (PP2C) superfamily: Detection of bacterial homologues. Protein Sci..

[170] Zhang H., Shi L., Li L., Guo S., Zhang X., Yamasaki S., Miyoshi S., Shinoda S. (2004). Identification and characterization of class 1 integron resistance gene cassettes among *Salmonella* strains isolated from healthy humans in China. Microbiol. Immunol..

[171] Shi L. (2004). Manganese-dependent protein O-phosphatases in prokaryotes and their biological functions. Front. Biosci..

[172] Das A.K., Helps N.R., Cohen P.T., Barford D. (1996). Crystal structure of the protein serine/threonine phosphatase 2C at 2.0 A. resolution. EMBO J..

[173] Alzari P.M. (2004). First structural glimpse at a bacterial Ser/Thr protein phosphatase. Structure.

[174] Pullen K.E., Ng H.L., Sung P.Y., Good M.C., Smith S.M., Alber T. (2004). An alternate conformation and a third metal in PstP/Ppp, the *M. tuberculosis* PP2C-Family Ser/Thr protein phosphatase. Structure.

[175] Shi L., Potts M., Kennelly P.J. (1998). The serine, threonine, and/or tyrosine-specific protein kinases and protein phosphatases of prokaryotic organisms: A family portrait. FEMS Microbiol. Rev..

[176] Zhang W., Shi L. (2004). Evolution of the PPM-family protein phosphatases in *Streptomyces*: Duplication of catalytic domain and lateral recruitment of additional sensory domains. Microbiology.

[177] Lu G., Wang Y. (2008). Functional diversity of mammalian type 2C protein phosphatase isoforms: New tales from an old family. Clin. Exp. Pharmacol. Physiol..

[178] Wehenkel A., Bellinzoni M., Schaeffer F., Villarino A., Alzari P.M. (2007). Structural and binding studies of the three-metal center in two mycobacterial PPM Ser/Thr protein phosphatases. J. Mol. Biol..

[179] Rantanen M.K., Lehtiö L., Rajagopal L., Rubens C.E., Goldman A. (2007). Structure of *Streptococcus agalactiae* serine/threonine phosphatase. The subdomain conformation is coupled to the binding of a third metal ion. FEBS J..

[180] Schlicker C., Fokina O., Kloft N., Grüne T., Becker S., Sheldrick G.M., Forchhammer K. (2008). Structural analysis of the PP2C phosphatase tPphA from *Thermosynechococcus elongatus*: A flexible flap subdomain controls access to the catalytic site. J. Mol. Biol..

[181] Madec E., Laszkiewicz A., Iwanicki A., Obuchowski M., Séror S. (2002). Characterization of a membrane-linked Ser/Thr protein kinase in *Bacillus subtilis*, implicated in developmental processes. Mol. Microbiol..

[182] Jiang S.M., Cieslewicz M.J., Kasper D.L., Wessels M.R. (2005). Regulation of virulence by a two-component system in group B *Streptococcus*. J. Bacteriol..

[183] Lamy M.C., Zouine M., Fert J., Vergassola M., Couve E., Pellegrini E., Glaser P., Kunst F., Msadek T., Trieu-Cuot P. (2004). CovS/CovR of group B *Streptococcus*: A two-component global regulatory system involved in virulence. Mol. Microbiol..

[184] Gryllos I., Grifantini R., Colaprico A., Jiang S., Deforce E., Hakansson A., Telford J.L., Grandi G., Wessels M.R. (2007). Mg(2+) signaling defines the group A streptococcal CsrRS (CovRS) regulon. Mol. Microbiol..

[185] Bugrysheva J., Froehlich B.J., Freiberg J.A., Scott J.R. (2011). Serine/threonine protein kinase Stk is required for virulence, stress response, and penicillin tolerance in *Streptococcus pyogenes*. Infect. Immun..

[186] Ulijasz A.T., Andes D.R., Glasner J.D., Weisblum B. (2004). Regulation of iron transport in *Streptococcus pneumoniae* by RitR, an orphan response regulator. J. Bacteriol..

[187] Chien Y., Manna A.C., Projan S.J., Cheung A.L. (1999). SarA, a global regulator of virulence determinants in *Staphylococcus aureus*, binds to a conserved motif essential for Sar-dependent gene regulation. J. Biol. Chem..

[188] Cheung A.L., Nishina K., Manna A.C. (2008). SarA of *Staphylococcus aureus* binds to the *sarA* promoter to regulate gene expression. J. Bacteriol..

[189] Donat S., Streker K., Schirmeister T., Rakette S., Stehle T., Liebeke M., Lalk M., Ohlsen K. (2009). Transcriptome and functional analysis of the eukaryotic-type serine/threonine kinase PknB in *Staphylococcus aureus*. J. Bacteriol..

[190] Fournier B., Hooper D.C. (2000). A new two-component regulatory system involved in adhesion, autolysis, and extracellular proteolytic activity of *Staphylococcus aureus*. J. Bacteriol..

[191] Fournier B., Truong-Bolduc Q.C., Zhang X., Hooper D.C. (2001). A mutation in the 5′ untranslated region increases stability of *norA* mRNA, encoding a multidrug resistance transporter of *Staphylococcus aureus*. J. Bacteriol..

[192] Kaatz G.W., McAleese F., Seo S.M. (2005). Multidrug resistance in *Staphylococcus aureus* due to overexpression of a novel multidrug and toxin extrusion (MATE) transport protein. Antimicrob. Agents Chemother..

[193] Dworkin M. (1996). Recent advances in the social and developmental biology of the myxobacteria. Microbiol. Rev..

[194] Nariya H., Inouye S. (2006). A protein Ser/Thr kinase cascade negatively regulates the DNA-binding activity of MrpC, a smaller form of which may be necessary for the *Myxococcus xanthus* development. Mol. Microbiol..

[195] Xu W.L., Jeanjean R., Liu Y.D., Zhang C.C. (2003). *pkn22 (alr2502)* encoding a putative Ser/Thr kinase in the cyanobacterium *Anabaena* sp. PCC 7120 is induced by both iron starvation and oxidative stress and regulates the expression of *isiA*. FEBS Lett..

[196] Janczarek M. (2011). Environmental signals and regulatory pathways that influence exopolysaccharide production in rhizobia. Int. J. Mol. Sci..

[197] Kusebauch U., Ortega C., Ollodart A., Rogers R.S., Sherman D.R., Moritz R.L., Grundner C. (2014). *Mycobacterium tuberculosis* supports protein tyrosine phosphorylation. Proc. Natl. Acad. Sci. USA.

[198] Dorman C.J., Deighan P. (2003). Regulation of gene expression by histone-like proteins in bacteria. Curr. Opin. Genet. Dev..

[199] Aki T., Choy H.E., Adhya S. (1996). Histone-like protein HU as a specific transcriptional regulator: Co-factor role in repression of gal transcription by GAL repressor. Genes Cells.

[200] Oberto J., Nabti S., Jooste V., Mignot H., Rouviere-Yaniv J. (2009). The HU regulon is composed of genes responding to anaerobiosis, acid stress, high osmolarity and SOS induction. PLoS ONE.

[201] Sajid A., Arora G., Gupta M., Singhal A., Chakraborty K., Nandicoori V.K., Singh Y. (2011). Interaction of *Mycobacterium tuberculosis* elongation factor Tu with GTP is regulated by phosphorylation. J. Bacteriol..

[202] Marrakchi H., Lanéelle M.A., Daffé M. (2014). Mycolic acids: Structures, biosynthesis, and beyond. Chem. Biol..

[203] Manuse S., Fleurie A., Zucchini L., Lesterlin C., Grangeasse C. (2016). Role of eukaryotic-like serine/threonine kinases in bacterial cell division and morphogenesis. FEMS Microbiol. Rev..

[204] Squeglia F., Marchetti R., Ruggiero A., Lanzetta R., Marasco D., Dworkin J., Petoukhov M., Molinaro A., Berisio R., Silipo A. (2011). Chemical basis of peptidoglycan discrimination by PrkC, a key kinase involved in bacterial resuscitation from dormancy. J. Am. Chem. Soc..

[205] Foulquier E., Pompeo F., Freton C., Cordier B., Grangeasse C., Galinier A. (2014). PrkC-mediated phosphorylation of overexpressed YvcK protein regulates PBP1 protein localization in *Bacillus subtilis mreB* mutant cells. J. Biol. Chem..

[206] Soufi B., Kumar C., Gnad F., Mann M., Mijakovic I., Macek B. (2010). Stable isotope labeling by amino acids in cell culture (SILAC) applied to quantitative proteomics of *Bacillus subtilis*. J. Proteome Res..

[207] Kamei A., Yuasa T., Geng X., Ikeuchi M. (2002). Biochemical examination of the potential eukaryotic-type protein kinase genes in the complete genome of the unicellular cyanobacterium *Synechocystis* sp. PCC 6803. DNA Res..

[208] Zhang X., Zhao F., Guan X., Yang Y., Liang C., Qin S. (2007). Genome-wide survey of putative serine/threonine protein kinases in cyanobacteria. BMC Genomics.

[209] Kamei A., Yoshihara S., Yuasa T., Geng X., Ikeuchi M. (2003). Biochemical and functional characterization of a eukaryotic-type protein kinase, SpkB, in the cyanobacterium, *Synechocystis* sp. PCC 6803. Curr. Microbiol..

